# Tomato Roots Exhibit Development‐Specific Responses to Bacterial‐Derived Peptides

**DOI:** 10.1111/pce.70164

**Published:** 2025-09-05

**Authors:** Rebecca Leuschen‐Kohl, Robyn Roberts, Danielle M. Stevens, Ning Zhang, Silas Buchanan, Brooke Pilkey, Gitta Coaker, Anjali S. Iyer‐Pascuzzi

**Affiliations:** ^1^ Department of Botany and Plant Pathology and Center for Plant Biology Purdue University West Lafayette Indiana USA; ^2^ Department of Agricultural Biology Colorado State University Fort Collins Colorado USA; ^3^ Department of Plant Pathology University of California Davis California USA; ^4^ Boyce Thompson Institute for Plant Research and Plant Pathology and Plant‐Microbe Biology Section, School of Integrative Plant Science Cornell University Ithaca New York USA

**Keywords:** flagellin, flagelling‐sensing‐2 (SlFLS2), flagellin‐sensing‐3 (SlFLS3), pattern‐triggered immunity, SlCORE, *Solanum lycopersicum* (tomato)

## Abstract

To combat soilborne pathogens, roots activate pattern‐triggered immunity (PTI) through pattern‐recognition receptors (PRRs) that recognise microbe‐associated molecular patterns (MAMPs). Root PTI pathways can differ from their above‐ground counterparts and have been well‐characterised in the model plant *Arabidopsis thaliana* but are not well‐defined in crops. Gene repurposing coupled with differences in root tissues and root architecture in tomato species (*Solanum lycopersicum* and *S. pimpinellifolium*) led us to hypothesise that signalling pathways of *Solanaceous*‐specific PRRs diverge from canonical pathways. The objective of this study was to characterise PTI signalling pathways and responses (ROS, MAPK, gene expression, and growth inhibition) in roots of wild and domesticated tomatoes downstream of three immune receptors: the well‐conserved *SlFLS2* and the *Solanaeceous*‐specific *FLS3* and *CORE*. We find that *Solanum* root PTI responses are concentrated in early differentiating root regions compared to late differentiating regions or whole roots, and that *FLS3* and *CORE* signalling pathways are overlapping but distinct from each other and from *FLS2*. Although the early differentiating root region had strong PTI responses across *Solanum* cultivars and species, different genetic backgrounds varied in their response dynamics. Our results underscore the complexity of PTI signalling across species and highlight the developmental‐stage specificity of tomato root immunity.

## Summary statement

- Pattern‐triggered immune responses in roots are concentrated in the early differentiating regions in wild and domesticated tomatoes (*Solanum pimpinellifolium* and *S. lycopersicum*).
- Signalling components and phenotypic responses downstream of different cell‐surface immune receptors are distinct but overlapping and vary among tomato genetic backgrounds.

## 1 Introduction

Despite the importance of soil‐borne pathogens, which can cause over 50% yield loss in crops, our understanding of the mechanisms governing immunity in crop roots has lagged behind that of aboveground organs (Tsai et al. 2023; Chuberre et al. 2018). Like their aboveground counterparts, plant roots exhibit a multi‐layered defence system, comprised of pre‐formed barriers and induced defence responses. In one of the earliest induced defence responses, cell surface‐localised receptor proteins known as pattern‐recognition receptors (PRRs) recognise biochemical and proteinaceous signatures from a pathogen in the initial stages of invasion (Yuan et al. 2021; Yu et al. 2021). These signatures, also called microbe‐associated molecular patterns (MAMPs), are found throughout a range of microbes, from soil‐borne bacteria to foliar fungi (Miya et al. 2007; Wei et al. 2018; Luo et al. 2023). Recognition of MAMPs by PRRs activates intracellular defence signalling with co‐receptors and kinase cascades (Asai et al. 2002; Boudsocq et al. 2010; Li et al. 2014; Lee et al. 2021), leading to a suite of canonical defence responses. These include the short‐term formation of reactive oxygen species (ROS), an influx of calcium, changes to cytoskeleton organisation (Li and Staiger 2018), halted growth, and altered transcriptional reprogramming – altogether known as pattern triggered immunity, or PTI (Shu et al. 2023). Although the overall PTI pathways are similar between below and aboveground organs, roots can differ in their immune responses compared to above‐ground counterparts, particularly in *PRR* gene expression (Wyrsch et al. 2015).

PTI pathways have been well‐characterised in roots of the model plant *Arabidopsis thaliana* (Arabidopsis) through studies of PRRs like the leucine‐rich repeat receptor‐like kinase FLAGELLIN SENSING2 (FLS2), which recognises a 22 amino acid fragment of flagellin known as flg22 (Gómez‐Gómez and Boller 2000; Chinchilla et al. 2006). In contrast, root PTI pathways are less well‐characterised in crop plants (Hudson et al. 2024). Interspecies transfer of a handful of Arabidopsis PRRs has successfully expanded crop resistance to some pathogens (Lacombe et al. 2010; Kunwar et al. 2018; Lu et al. 2015; Mitre et al. 2021 (Frailie and Innes 2021), but has also faced challenges, potentially due to divergences in coreceptor requirements (Yang et al. 2023; Yang et al. 2025) or downstream signalling pathways (Yang et al. 2025). Thus, while PRR‐based crop engineering has the potential to provide broad‐spectrum and durable resistance (Lacombe et al. 2010; Li et al. 2024), detailed knowledge of PTI in crops is needed (Hudson et al. 2024). This is particularly true in plant roots, which are composed of distinct cell types in different developmental stages and can vary in architecture and tissue types across species (Kajala et al. 2021; Huang et al. 2017; Canto‐Pastor et al. 2024). Compared to Arabidopsis, tomato roots have differences in cortex layer number, transcription factors promoting xylem differentiation, root angle formation, lateral root formation, and root hair patterning (Tominaga‐Wada et al. 2013; Kajala et al. 2021; Huang et al. 2017). Further, the tomato genome encodes additional PRRs that expand MAMP recognition in this species (Hind et al. 2016; Wang et al. 2016). Given these differences, we hypothesised that the molecular details of canonical PTI signalling may differ in tomato roots downstream of *Solanum*‐specific PRRs compared to well‐conserved PRRs.

Our objective was to characterise PTI signalling pathways from receptor recognition to transcriptional output for different PRR‐MAMP combinations in roots of wild (*Solanum pimpinellifolium*) and domesticated tomatoes (*Solanum lycopersicum*). We focused on three PRRs: *SlFLS2*, the tomato homologue of Arabidopsis *FLAGELLIN SENSING 2* that recognises a 22 amino acid epitope of bacterial flagellin, *FLAGELLIN SENSING3* (*FLS3*), the receptor for another flagellin‐derived epitope, flgII‐28 (Felix and Boller 2003; Hind et al. 2016) and *CORE*, the receptor for cold shock protein 22 (csp22) (Wang et al. 2016). *FLS2* is a broadly conserved PRR across plant genomes (Cheng et al. 2020), while *FLS3* and *CORE* have been identified in a subset of *Solanaceous* genomes but have not been identified outside of *Solanaceous* species (Hind et al. 2016; Wang et al. 2016).

Using these receptors and their MAMPs, we investigate PTI signalling and responses in different accessions of domesticated tomato (*S. lycopersium*) and in wild tomato (*S. pimpinellifolium*) roots, tracing the pathway from cell surface recognition to downstream phenotypic outcomes. We find that the tomato root PTI response is primarily found in the early differentiating regions of both wild and domesticated tomato roots for all three immune receptors. While ROS burst occurs downstream of each immune receptor, the amplitude and dynamics varies by receptor and tomato genotype. Intermediate signalling components such as receptor‐like cytoplasmic kinases (RLCKs), MAPK phosphorylation, and transcriptional output differ among *SlFLS2*, *FLS3* and *CORE*. Finally, responses such as temporary root growth inhibition diverge between *SlFLS2* compared to *FLS3* and *CORE*. Our results highlight the variation among molecular signalling in root PTI pathways among different PRR receptors as well as within plant species and call attention to the need to understand the diversity of PTI responses to develop crops with increased disease resistance.

## 2 Materials and Methods

### 2.1 Plant Material and Plate Growth Conditions

Tomato accessions listed in Table 1 were sterilised for 10 min in 50% bleach, then washed three times with water. Seeds were plated on 1% agar plates at 4°C overnight before placing at room temperature (22°C) at a 16:8 h day/night cycle.

**Table 1:** Tomato accessions used in this study.

Species	Cultivar	Notes	Reference
*Solanum lycopersicum*	H7996		
	Yellow Pear	No *FLS3* expression	Hind et al. (2016)
	Brandywine		
	Black from Tula		
	Ailsa Craig		
	Rutgers		
	Rio Grande		
	Rio Grande *PtoR*		
	*rbohb* (Rio Grande *PtoR* background)	*RbohB* genome edited line	Figure S9
*Solanum pimpinellifolium*	LA2093		
	Wv700		
*Solanum pennellii*	LA0716	No *CORE* expression	Wang et al. (2016)

*Arabidopsis thaliana* seeds (Col‐0, *AtrbohD, AtrbohF, AtrbohD/AtrbohF*) were sterilised for 5 min in 50% bleach and 0.001% Tween, then washed three times with water. Seeds were stratified in ddH20, then covered for 48 h at 4° C before plating on 0.5X Murashige and Skoog (MS) medium, 1% sucrose. Seeds were grown in a controlled chamber at 22°C at a 16:8 h day/night cycle. Mutant seeds were obtained from the lab of Chris Staiger, Purdue University Department of Botany and Plant Pathology.

### 2.2 Generation of *rbohb* Mutant in Tomato

Mutant seeds (Rio Grande PtoR – *SlrbohB; referred to rbohb thereafter*) were generated using genome editing approaches as previously described in Zhang et al. (2020). To generate the *rbohb* mutant in the tomato (*Solanum lycopersicum*) accession Rio Grande (RG)‐ PtoR, one guide RNA (gRNA: 5′‐ GGACCGCTGAACAAACGAGG‐3′) was designed to target the first exon of *RbohB* (Solyc03g117980). The gRNA cassette was cloned into the p201N:Cas9 binary vector and tomato transformation was performed at the Biotechnology Center at the Boyce Thompson Institute as described previously (Jacobs et al. 2015; Jacobs et al. 2017). The *rbohb* mutant line used in this study carries a 1 bp insertion in the first exon of the *SlRbohB* gene, resulting in a loss‐of‐function mutation in *SlRbohB* in the plants. Mutations were confirmed by PCR amplification using primers found in Table S1 and Sanger sequencing. Lines were verified to be homozygous, knockout mutants and Cas9 was segregated out.

### 2.3 Peptides

flg22^Pst^ and csp22 peptides were purchased from EZBiolabs, using the following amino acid sequences: flg22^Pst^ QRLSTGSRINSAKDDAAGLQIA; csp22^Rsol^: ATGTVKWFNETKGFGFITPDGG.

The flgII‐28^Pst^ and flg22^Rsol^ peptide was purchased from GenScript, with the following amino acid sequence: flgII‐28^Pst^: ESTNILQRMRELAVQSRNDSNSATDREA, flg22^Rsol^ QRLSTGLRVNSAQDDSAAYAAS.

### 2.4 Temporary Root Growth Inhibition (RGI) Assay

Tomato seedlings were grown on 1% water agar plates in the conditions as described above. Four‐day old seedlings were scanned and treated with 300 µL of elicitor treatment (1 µM flg22^Pst^, 100 nM flgII‐28^Pst^, 1 µM csp22^Rsol^, or water), making sure to only submerge the root organ. Tomato seedlings were then scanned again at 24‐ and 48‐h postinoculation and measured using ImageJ for subsequent analysis.

Arabidopsis seedlings were grown on 0.5× MS, 1% sucrose in the conditions as described above. Five‐day old seedlings were scanned and treated with 200 µL of elicitor treatment (1 µM flg22^Pst^ or water), making sure to only submerge the root organ. Arabidopsis seedlings were then scanned again at 24‐ and 48‐h postinoculation and measured using ImageJ for subsequent analysis.

### 2.5 Oxidative Burst Luminescence Assay

The ROS assay was performed on tomato roots as described previously with a number of modifications (Wei et al. 2018). For whole‐root assays, tomato seedlings were grown on 1% agar in the conditions described above. Five‐day old tomato roots were placed under microscope and cut at the root‐shoot junction. For developmental zone assays, the 5‐day old tomato roots were placed under a microscope and cut at the point of first visual root hair, the point at which root hairs had fully emerged, and at the root‐shoot junction. The early differentiation zone (ED) was defined as the root section exhibiting emerging root hairs, while the late differentiation zone (LD) exhibited fully emerged root hairs. All root segments were then weighed with a precision balance before being placed in a white 96‐well plate (Perkin Elmer, OptiPlate‐96) with 200 µL of fresh water to recover. Segments were washed with water and kept in the dark for 1 h, after which the water was removed, and fresh water was placed in each well and sat overnight in darkness. After overnight recovery, the water was removed and replaced with 200 µL of the corresponding master mix for each peptide elicitor. Master mix was made from 500× L‐012 stock solution (LSS) and 500× horseradish peroxidase stock solution (HPSS) and the corresponding peptide for a final concentration of 1.5× L‐012 (Wako Chemicals USA) and 1.5× HPSS (Thermo Fisher Scientific). Master mixes used had a final peptide concentration of 1 µM flg22^Pst^, 100 nM flgII‐28^Pst^, or 1 µM csp22^Rsol^. Relative light units (RLUs) were detected using an Infinite 200 Pro Luminescent Microplate Reader (Tecan Life Sciences, Switzerland) and exported to an excel spreadsheet for further analysis. Three technical replicates were used for each analysis, with six roots per treatment. Data were normalised and expressed as RLU per milligram of fresh weight.

For tomato leaves, ROS assays were performed as previously described in (Hind et al. 2016) using 100 nM of flg22^Pst^ or flgII‐28^Pst^ peptides. The average ROS response for each plant is the mean of three replicate leaf discs from four plants. The assay was performed on ten independent VIGS biological replicates with similar results, and one representative experiment is shown in Figure 3.

### 2.6 Cloning

Constructs for the VIGs assays were amplified via PCR using the primers found in Table S1. Total RNA was extracted from tomato leaves (Rio Grande) using the Qiagen RNeasy Plant Mini Kit (Cat. 74904) and used to generate cDNA (Invitrogen SuperScript III, 12574018).

### 2.7 Virus‐Induced Gene Silencing (VIGS)

The pTRV vector derivatives (pTRV2‐EC1, pTRV2‐SlSERK3A, pTRV2‐SlSERK3B, and pTRV2‐SlSERK3A/3B) were transformed into *Agrobacterium tumefaciens* strain GV3101and prepared for infection (final OD = 0.5) in tomato seedlings as previously described (del Pozo et al. 2004). Knockdown of gene expression in leaf tissues was confirmed in qPCR using the primers in Table S1 as described previously (Mantelin et al. 2011). VIGS experiments were repeated a total of ten times using four plants per replicate (*n* = 40 for each VIGS construct) with similar results.

### 2.8 Treatments of Diphenyleneiodonium Chloride (DPI)

To determine the concentration of diphenyleneiodonium chloride (Sigma Aldrich, CAS: 4673‐26‐1) required to inhibit ROS burst caused by flg22^Pst^, the oxidative burst luminescence assay above was repeated with mock, 1 μM flg22^Pst^, and 1uM flg22^Pst^ solutions containing a final concentration of DPI between 0 and 1 μM.

Root growth assays including DPI were treated 1 h before inoculation with 1 μM DPI as determined by the oxidative burst luminescence assay referenced above. The roots were then treated at 0 hpi with an elicitor solution of mock, 1 μM flg22^Pst^, or 1 μM flg22^Pst^ and 1 μM DPI.

### 2.9 Plant Growth of Tomato Accessions in Soil

H7996 (*S. lycopersicum*) was sterilised using the above method. Seeds were stratified in water and left at 4C overnight before planting. Plants were grown in conditions as described in Meline et al. (2023) with slight modifications. Seeds were grown in BM3 in 3.8 cm × 8.6 cm × 5.8 cm (L × W × D) at 28°C and 16/8 h day/night. Twelve days after germination, plants were treated with 28 mL of Peter's Excel Fertiliser (86.4 g/L).

### 2.10 Determination of MAPK Phosphorylation

Tomato (H7996) 5‐day old seedlings were cut from the above‐ground tissues at the root‐shoot junction and further separated into whole root samples, late differentiation zone samples, or early differentiation zone samples. The root segments were allowed to sit for 6 h in ddH_2_0 before being placed into a solution of 1 µM flg22^Pst^, 100 nM flgII‐28^Pst^, or 1 µM csp22^Rsol^. The tissue was harvested at 0‐ or 10‐min post treatment and flash frozen in liquid nitrogen. For tomato leaves, leaf discs were collected from 8‐week‐old tomato leaves (H7996) and allowed to sit for 6 h before being placed into a solution of 1 µM flg22^Pst^, 100 nM flgII‐28^Pst^, or 1 µM csp22^Rsol^ and flash frozen in liquid nitrogen after 10 min.

Total proteins were extracted using a protein extraction buffer (50 mM Tris‐HCl [pH 7.5], 150 mM NaCl, 0.1% Triton X‐100) containing 1% protease inhibitor cocktail (here) and 1% Phosphatase Inhibitor Cocktail 2 (Sigma‐Aldrich, P5726). After extraction, total protein was incubated with 4X Laemmli SDS Buffer (Fisher Scientific) and heated for 10 min at 95°C. Proteins were separated by SDS‐PAGE (10% acrylamide) and were transferred to a nitrocellulose membrane. After blocking with 1% BSA in TBS‐Tween (0.01%) buffer for 1 h at room temperature. Phosphorylation of MAP Kinases were detected by an antiphospho‐p44/42 MAPK (Erk1/2) (Thr202/Tyr204) HRP‐conjugated antibody (Cell Signalling Technology) and actin was detected by HRP conjugated Anti‐Plant Actin Mouse Monoclonal Antiboty (3T3) (Abbkine, ABL1055). Signals were detected using SuperSignal West Pico Plus Chemiluminescent Substrate (Thermo Fisher). MAPK activation was quantified using an established ImageJ plugin (Ohgane and Yoshioka 2019).

### 2.11 Total RNA Extraction for RNA‐Seq of Tomato Roots

Five‐day‐old H7996 seedlings were cut into whole root, late differentiation, and early differentiation zones using the same methods as the ROS and MPK assays. The root segments were left in water overnight to recover and then treated with 1 uM flg22^Pst^, 100 nM flgII‐28, or mock water. Six root samples from each segment type and treatment were pooled at 6 h postinoculation, and the samples were ground into a powder using a mortar and pestle under liquid nitrogen. Whole root and LD samples (100 mg ± 10) or ED samples (20 mg ± 5) of root ground tissue from each sample was used for RNA extraction using Trizol (Invitrogen), following the manufacturer's instructions. RNA purification was done with Qiagen RNeasy mini‐Kit with DNase I treatment in‐column treatment.

### 2.12 RNA‐Seq

Three biological replicates (each consisting of roots from three individual plants) per accession and treatment were subjected to Illumina RNA sequencing. Each sample averaged about 45.7 million (range from 27.1 to 66.6 million) high quality paired end reads. More than 94% of the reads were mapped to the ITAG4.1 *Solanum lycopersicum* reference genome, using STAR version 2.7.10.a. Gene expression was measured as the total reads for each sample that uniquely mapped to the reference gene list with summarizeOverlaps (GenomicAlignments1.34.1 and Rsamtools 2.14.0). Data was filtered for low counts such that at least three of the 12 samples had at least three counts per row. Differential gene expression analysis was performed with DESeq. 2 version 1.38.3. We used an FDR < 0.05 to determine differentially expressed genes. Gene ontology (GO) and KEGG analysis were performed using ShinyGo 0.80 for categories that contained less than 500 terms in their corresponding category. Heatmaps were visualised with R software version 3.4.0 package “ggplot2”.

### 2.13 Statistical Analyses

Statistical analyses were conducted in R version 3.4.0. Data distribution was assessed, and tests appropriate to the distribution of the data were applied.

## 3 Results and Discussion

### 3.1 Tomato Root ROS Response to MAMPs Is Primarily Located in the Early Differentiation Zone

**Figure 1:**
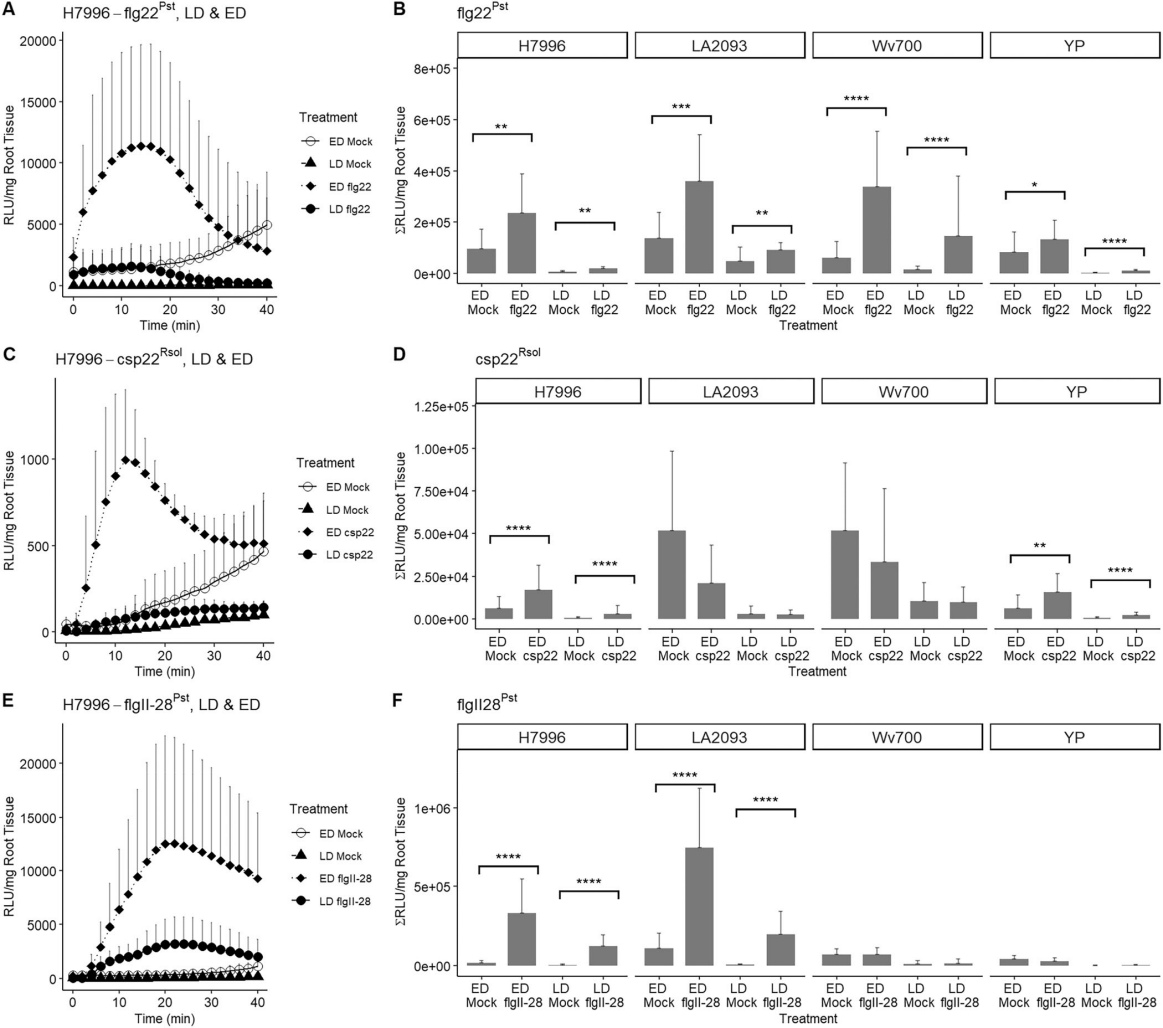
Reactive oxygen species burst is primarily found in the early differentiation zone. Reactive oxygen species burst is primarily found in the early differentiation zone. Root samples of the LD and ED Zone from 5‐day‐old tomato seedlings. (A) RLU over time for H7996, and (B) Total RLU for H7996, LA2093, Wv700, and Yellow Pear root samples treated with 1 µM flg22^Pst^ or mock (water). (C) RLU over time for H7996 and (D) Total RLU for H7996, LA2093, Wv700, and Yellow Pear treated with 1 µM csp22^Rsol^ or mock (water). (E) RLU over time for H7996, and (F) Total RLU for H7996, LA2093, Wv700, and Yellow Pear treated with 1 µM flgII‐28^Pst^ or mock (water). For each PAMP, the ROS peak was highest in the ED zone. Values in Figure 1B,D,F represent the mean ± SD from at least sixreplicates per treatment. The assay was repeated three times with similar results. Values in Figure 1C,E,G represent the mean + SD from at least 18 replicates per treatment (Student's *t*‐test, *p < 0.05, **p < 0.01, ***p < 0.001, ****p < 0.0001).

Tomato roots are most sensitive to soil borne pathogens like *Ralstonia solanacearum* in the late elongation and differentiation zone (Vasse 1995; Kashyap et al. 2022). We hypothesised that PTI responses would be highest in tomato roots in these zones. We first established a root ROS assay with various *S. lycopersicum* and *S. pimpinellifolium* accessions (Table 1) and 1 µM flg22^Pst^. As expected, given expression of *FLS2*, whole roots of all accessions responded to flg22^Pst^ (Figure S1A) but not the flg22 peptide from *R. solanacearum*, which is not recognised by FLS2 (Figure S1B). To test for development‐dependent PTI responses in tomato roots, tomato primary roots were cut into sections, including the early differentiation (ED) Zone and the Late Differentiation (LD) Zone. The ED Zone was characterised by the presence of visible emerging root hairs, while the LD Zone exemplified fully emerged root hairs on the primary root (Figure S2A). Treating each zone with flg22^Pst^ revealed that the ED Zone was most responsive across all accessions (Figure 1A,B).

To test whether the ED Zone had consistently higher ROS than the LD Zone for other MAMP treatments, we tested the effect of two additional MAMPs: csp22^Rsol^ and flgII‐28^Pst^. Similar to flg22^Pst^, treatment with 1 µM csp22 resulted in an increase in ROS in the ED zone compared to the LD Zone (Figure 1C,D) in *S. lycopersicum* accessions H7996 and YP, however not all accessions responded to csp22^Rsol^. The *S. pimpinellifolium* LA2093 and Wv700 accessions did not respond (Figure 1D). *S. pennellii* accession LA0716, which lacks expression of *SlCORE* (Wang et al. 2016), was used as a control for our csp22^Rsol^ peptide (Figure S3A) and also showed no response. The CORE receptor has age‐dependent expression in leaves of *N. benthamiana* (Dodds et al. 2023) and it was possible that the receptor was not expressed in either of the root developmental zones we tested in these accessions. Thus, we tested the whole root response to csp22 in each tomato accession. Like our developmental zone results, whole roots of H7996 responded to csp22^Rsol^ while Wv700 did not respond, suggesting that Wv700 does not express the *CORE* receptor in young roots. However, in contrast to our developmental zone results, the whole roots of LA2093 responded to csp22^Rsol^ while YP did not (Figure S1C). It is possible that expression of *CORE* in LA2093 is not sufficiently high in the ED or LD zones to elicit a ROS burst. In Yellow Pear, the ROS burst is confined to the ED zone but is significantly lower than for flg22^Pst^ (compare 1E to 1C). The small ROS burst in Yellow Pear may explain why a burst is not observed in the whole root data. Therefore, we tested whether the ED zone was the primary site of csp22 response in four additional *S. lycopersicum* accessions. Accessions Brandywine, Rutgers, and Ailsa Craig also showed significant ROS burst in the ED zone compared to the LD; Black from Tula, however, showed a prominent ROS response in both the ED and LD zones (Figure S2B–E). Together, these data suggest that accessions may show accession‐specific distribution of CORE. However, characterisation of older roots ( > 6 weeks) is needed to further understand CORE‐mediated ROS response in belowground tissues.

We next tested flgII‐28, which is perceived by the receptor FLS3 (Hind et al. 2016). Because 1 µM flgII‐28 elicits a significantly stronger ROS burst compared to 1 µM flg22^Pst^ in tomato leaves (Zeiss et al. 2021) and in potato leaves and root tips (Moroz and Tanaka 2020), we first tested two concentrations in roots of H7996 (*S. lycopersicum*) and LA2093 (*S. pimpinellifolium*): 100 nM and 1 µM. Both concentrations elicited a stronger ROS burst (Figure S4A,B) in the ED Zone compared to the LD Zone. The ROS burst in the ED zone elicited by both flgII‐28^Pst^ concentrations was higher than that observed for 1 µM flg22^Pst^ (2X the flg22^Pst^ burst for 100 nM flgII‐28^Pst^ and ~3X for 1 uM flgII‐28^Pst^). We elected to use the smaller dose, 100 nM, for further assays with flgII‐28^Pst^ because this ROS burst was closer to that of 1 µM flg22^Pst^. We also reasoned that a more similar response may reveal species‐specific differences. We treated the ED zone and LD zone (Figure 1E,F) or whole roots (Figure S1D,E) of all four accessions with flgII‐28. As expected for YP, which does not express *FLS3* (Hind et al. 2016), there was no response in either the whole root or the ED (Figure 1F, S1D). H7996 and LA2093 responded highest in the ED zone compared to the LD. Similar to CORE, Wv700 did not show a response in any zone, suggesting that this accession of *S. pimpinellifolium* does not express *FLS3*. This is consistent with previous transcriptional data from Wv700 showing no expression of *CORE* or *FLS3* in whole roots of 3 and 4 week old plants (French et al. 2018; Meline et al. 2023).

### 3.2 SERK3A Is Primarily Responsible for FLS3‐Mediated ROS Burst

Although all three MAMPs elicited a ROS burst in the H7996 tomato roots, the dynamics of the burst varied. Therefore, we hypothesised that the downstream signalling pathway may differ among receptors. Due to the limited csp22^Rsol^ burst, we focused on FLS2 and FLS3 responses. We first concentrated on understanding the involvement of their co‐receptors, *Sl*SERK3a and *Sl*SERK3b, which are orthologs of Arabidopsis BAK1 (Peng and Kaloshian 2014). Tomatoes silenced for *Sl*SERK3a, *Sl*SERK3b, or both, show a severe reduction in SlFLS2‐mediated ROS production (Peng and Kaloshian 2014), and it has been shown that SERK3b, but not SERK3a, is phosphorylated upon activation of tomato PRR *Sl*PORK1 (Cho et al. 2024). However, the requirement of *Sl*SERK3a and *Sl*SERK3b for FLS3 is unknown.

**Figure 2:**
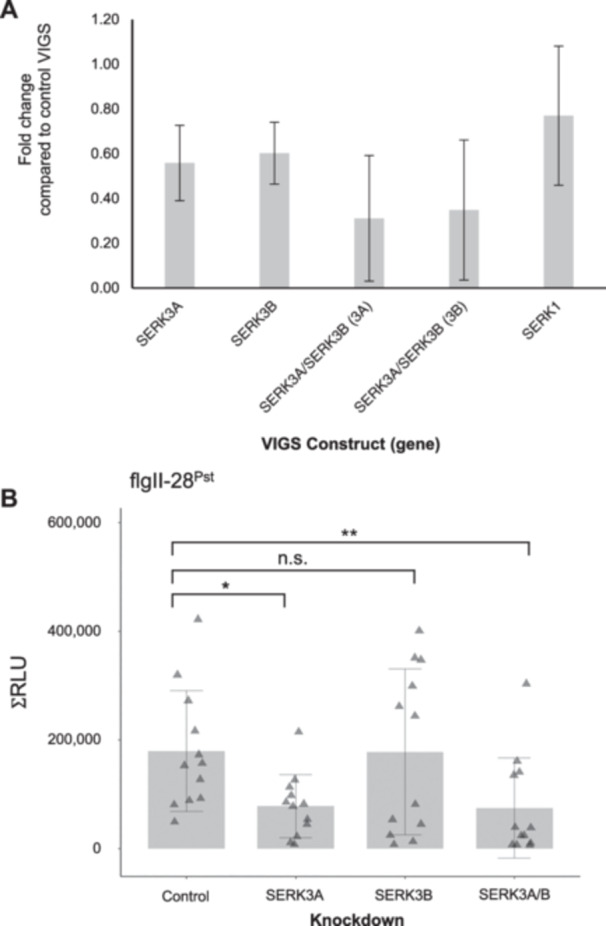
SERK3A and SERK3B are differentially required for flagellin PRRs FLS2 and FLS3. (A) qPCR of virus‐induced gene silencing (VIGS) constructs confirming reduced expression. Shown is the fold change relative to the expression of the associated gene in the empty control (EC1) VIGS. Four technical replicates (*n* = 4) and two biological replicates were performed for each VIGS‐silenced construct and confirmed for four of the ten VIGS biological replicates with similar results. The standard deviation (*n* = 4) is represented with error bars. The figure shows a single VIGS replicate. (B) Total ROS produced through addition of 100 nM flgII‐28^Pst^ in tomato accession Rio Grande when genes *SERK3A*, *SERK3B*, or both *SERK3A* and *SERK3B* (*SERK3A/3B*) are knocked down using virus‐induced gene silencing (VIGS) alongside the empty control (EC1). The figure shows one representative replicate (*n* = 4 plants of each VIGS). The experiment was repeated ten times with similar results (*n* = 40). (Mann‐Whitney U Test, *p < 0.05, **p < 0.01, ***p < 0.001, ****p < 0.0001).

To investigate the roles of SERK3A and SERK3B in detecting flgII‐28^Pst^, the expression of tomato orthologs *SERK3A*, *SERK3B*, and both *SERK3A* and *SERK3B* (*SERK3A/SERK3B*) were knocked down in *S. lycopersicum* leaves using virus‐induced gene silencing and knockdown in expression was confirmed using qPCR (Figure 2A). While H7996 roots were used for all other phenotypic and molecular experimentation due to its response to all three MAMPs, it was not receptive to extensive transformation efforts. Therefore, Rio Grande, an accession with established transformation protocols (Jacobs et al. 2017; Zhang et al. 2020), was used for the transformation studies (Hind et al. 2016). Rio Grande also showed ROS burst for flg22^Pst^ and flgII‐28^Pst^ in leaves (Roberts et al. 2019, 2020; Veluchamy et al. 2014) and roots (Figure S5).

Leaves silenced for *SERK3A* or for both *SERK3A/3B* and treated with 100 nM flgII‐28^Pst^ (Figure 2B) had a significant reduction in the total amount of ROS compared to the control vector after exposure to flgII‐28^Pst.^ In contrast, the *SERK3B* knockdown was not significantly different than the control. Previous results from Peng and Kaloshian (2014) showed that knocking down either *SERK3A* or *SERK3B* resulted in ROS burst reduction after flg22^Pst^ exposure. Together, these data suggest differences in coreceptor use by FLS2 and FLS3. SERK3A is necessary and sufficient for immunity activation by FLS3, while SERK3B does not appear to be required for FLS3‐activated immune ROS responses.

### 3.3 PTI Driven MPK Activation Is PRR‐Specific and Is Primarily Located in the Early Differentiation Zone

**Figure 3:**
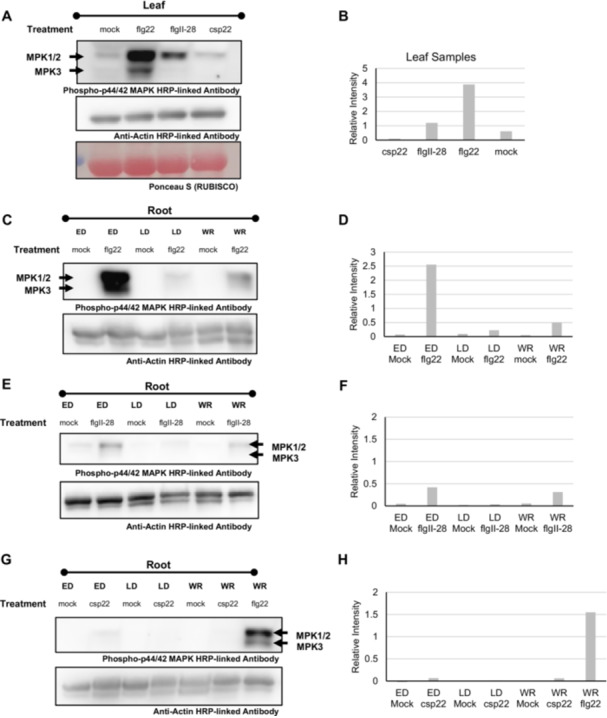
MPK phosphorylation in tomato leaf and root tissues of H7796 upon treatment with various MAMPs. (A) Eight‐week‐old leaf samples treated with mock (water), 1 µM flg22^Pst^, 100 nM flgII‐28^Pst^, or csp22 for 10 min. (B) Quantification of MAPK phosphorylation in leaf samples from 4a, normalised to actin. (C) Root sections representing LD, ED, and WR treated with mock (water) or 1 µM flg22^Pst^. (D) Quantification of MAPK phosphorylation in root sections from 4c, normalised to actin. (E) Root sections treated with mock (water) or 100 nM flgII‐28^Pst^. (F) Quantification of MAPK phosphorylation in root sections from 4e, normalised to actin. (G) Root sections treated with mock (water) or csp22^Rsol^. (H) Quantification of MAPK phosphorylation in root sections from 4 g, normalised to actin. Phosphorylation was assessed by western blot using Phospho‐ERK1/2 HRP‐linked antibody (CellSignaling, #8544). Total proteins were detected by Anti‐Actin HRP‐linked Antibody (Abbkine). A Bradford assay was also used for equal protein loading. The assay was repeated three times with similar results. [Color figure can be viewed at wileyonlinelibrary.com]

Given differences in coreceptor requirements between SlFLS2 and FLS3, we next focused on mitogen‐activated protein kinases (MAPK/MPKs) as these proteins are critical signalling components downstream of PRRs. SlMPK1/2/3, homologues of Arabidopsis MAPK3/MAPK6, are signalling proteins downstream of SlFLS2 in the tomato immune pathway (Pedley and Martin 2004; Stulemeijer et al. 2007; Willmann et al. 2014). To test whether these signalling proteins were conserved downstream of FLS3 and CORE, we first observed MPK1/2/3 phosphorylation of 8‐week‐old leaf tissue in H7996 upon treatment with flg22^Pst^, flgII‐28^Pst^ or csp22^Rsol^. As expected, flg22^Pst^ treatment resulted in activation of both MPK1/2 (45 kDa) and MPK3 (42 kDa) 10 min after treatment (Figure 3A,B) (Pedley and Martin 2004). In contrast, flgII‐28^Pst^ exhibited MPK phosphorylation for MPK1/2, but not MPK3, and treatment with csp22^Rsol^ did not result in phosphorylation (Figure 3A,B). Wei et al. (2018) reported a delayed MAPK response to csp22^Rsol^ in *N. benthamiana* leaves. Therefore, we tested whether MPK phosphorylation after csp22^Rsol^ treatment occurred at a later time point. We repeated the experiment, this time collecting samples at 10, 20, and 30 min. In leaves, flg22^Pst^ MPK1/2/3 phosphorylation was present at 20 min, whereas there was no MPK phosphorylation at 10, 20, or 30 min for samples treated with csp22^Rsol^ (Figure S6A). Together these data show that signalling downstream of FSL2, FLS3 and CORE differs in tomato, either in dynamics, intensity, or both.

We next asked whether MPK1/2/3 phosphorylation occurred in the root, and if so, whether it followed our ROS burst data and primarily occurred in the ED zone. We observed MPK phosphorylation of the ED, LD and whole root segments 10 min after treatment with 1 µM flg22^Pst^, 100 nM flgII‐28^Pst^ or 1 µM csp22^Rsol^ in 5‐day old tomato seedlings. Consistent with our ROS data, the ED Zone showed heightened MPK phosphorylation when compared to the LD Zone or whole root for both flg22^Pst^ and flgII‐28^Pst^ treatment. Treatment with flg22^Pst^ elicited phosphorylation of both MPK1/2 and MPK3 (Figure 3C). In contrast, flgII‐28^Pst^ treatment elicited phosphorylation primarily of MPK1/2 rather than MPK3 (Figure 3C–F). While it is possible that the MPK3 is present in roots but too low to be detected, FLS3 signalling in *Solanum tuberosum* also activates primarily SlMPK1/2 (Moroz and Tanaka 2020).

Similarly to the ED ROS data for csp22^Rsol^, the strength of the PTI response was far lower, if not absent, in tomato ED, LD, and WR sections (Figure 3G‐H). As with the leaf tissue, we tested whether the dynamics of MPK phosphorylation upon csp22^Rsol^ treatment were delayed in tomato roots and observed little‐to‐no MPK phosphorylation for roots at any time point (Figure S6B). In parallel with the developmental‐specificity of PTI‐driven ROS burst, these data support the ED zone as the primary location for PTI initiation and response.

The mechanisms driving differential phosphorylation of MAPK1/2/3 in tomato PTI remain unclear, but could be due to differences in signalling components that act downstream of the activated PRR complex but upstream of MPK cascades. Receptor‐like cytoplasmic kinases (RLCKs) are likely candidates for these differences. Unlike Arabidopsis, where AtBIK1 serves as a central regulator for PTI responses, functional divergence has resulted in no direct homologue of AtBIK1 in tomato, although a handful of RLCKs in the tomato genome interact with SlFLS2 and SlFLS3. Tomato RLCKs involved in flagellin‐derived PTI responses are not well characterised beyond that of SlTPK1b (functions downstream of FLS2) and SlFIR1 (functions downstream of both FLS2 and FLS3) (Abuqamar et al. 2008; Sobol et al. 2023). Mutations in SlFIR1 exhibit lower levels of ROS in leaves upon treatment with both flg22 and flgII‐28 (Sobol et al. 2023), but no change to levels of MPK phosphorylation. The signalling events downstream of SlFIR1 and SlTPK1b are not fully understood, and such components mediating tomato PRR‐specific responses remain a largely unexplored area of Solanaceous PTI.

### 3.4 Transcriptional Reprogramming After Mamp Treatment Is Heightened in the ED Zone

Pattern triggered immunity leads to changes in gene expression in roots, including that of increased PRR expression (Rich‐Griffin et al. 2020; Poncini et al. 2017, Millet et al. 2010). Therefore, we reasoned that the ED Zone would have a heightened transcriptional biotic stress response after MAMP treatment compared to the LD zone and whole roots. To examine this, we used root sections of H7996 treated with flg22^Pst^ or flgII‐28^28Pst^–as both MAMP treatments showed ROS and MPK responses in the root tissues (Figure 1A,B,E,F and Figure 3C–F). The whole root, LD, and ED sections were cut, washed, and left overnight before MAMP or water treatment (Modified from Wei et al. 2018). At 6 h posttreatment, roots were collected for RNA extraction, sequencing, and subsequent analysis using DESeq. 2 for identification of differentially expressed genes (DEGs).

**Figure 4:**
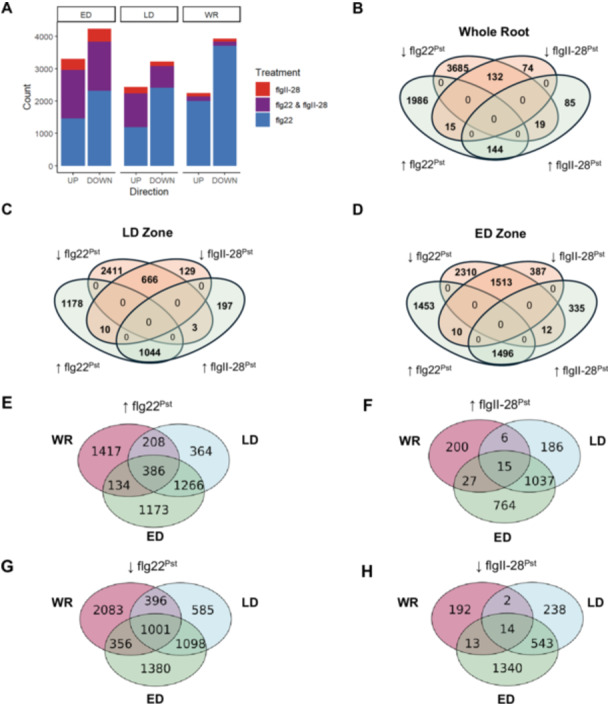
Differentially expressed genes in H7996 6 h after treatment with 1 μM flg22 or 100 nM flgII‐28 compared to mock treatment. (A) Stacked bar plot representing the count of total up or downregulated genes in each root zone for roots treated with flg22, flgII‐28, or both flg22 and flgII‐28. Venn diagram depicting both up‐ and downregulated DEGs for (B) whole root, (C) Late Differentiation zone, or (D) Early Differentiation zone samples after treatment with flg22^Pst^ or flgII‐28^Pst^. Overlap in (E) upregulated and (F) downregulated DEGs for Whole Root, Late Differentiation, and Early Differentiation Zone samples treated with flg22^Pst^. Overlap in (G) upregulated and (H) downregulated DEGs for Whole Root, Late Differentiation, and Early Differentiation Zone samples treated with flgII‐28^Pst^. DESeq. 2, *p*‐adj < 0.05. [Color figure can be viewed at wileyonlinelibrary.com]

Consistent with our ROS and MAPK data, more DEGs were identified in the ED zone for each peptide treatment compared to mock in the whole root and LD (Figure 4A–D). Notably, in the flgII‐28^Pst^ whole root samples only 248 genes were upregulated, and 221 genes were downregulated compared to the ED zone's 1843 and 1910 genes, respectively (Figure 4B vs. Figure 4D). In addition, the majority of the DEGs found in the ED samples were not identified in our whole root or LD samples (Figure 4E–H). The identification of genes distinctly upregulated in the ED shows that transcriptional regulation in the whole root is not reflective of the ED response and is consistent with our data showing the ED exhibits a distinct PTI response.

To more accurately understand the function of the DEGs found in our analysis, we performed a GO Biological Process analysis with the ShinyGO toolkit 0.80 within the ED, LD and whole root. More GO categories involved in plant‐microbe responses were present in the top 20 categories for the ED zone compared to the LD zone and whole root for both MAMP treatments (Figures S7, S8). For example, “Response to biotic stimulus” was among the top 20 GO categories in the ED zone but not in the whole root for both flg22^Pst^ and flgII‐28^Pst^. In addition, categories such as “cellular response to chemical stimulus” or “response to organic stimulus” had a higher number of DEGs found in the ED samples compared to the LD (Figure S7A,B vs. Figure S7C,D, orange arrows) for both peptide treatments. In addition, “response to biotic stimulus” was only in the top 20 GO categories for the ED zone treated with flg22^Pst^, but not the whole root (Figure S7A vs. S7C, green arrows)

We next investigated whether flg22^Pst^ and flgII‐28^Pst^ elicited similar transcriptional responses in the ED zone. More genes were differentially expressed after flg22^Pst^ treatment (SlFLS2‐response genes) compared to flgII‐28^Pst^ (FLS3‐response genes) (Figure 4A). Most of the FLS3‐response genes were also FLS2‐response genes (Figure 4A‐B). For example, in the ED zone, over 81% of FLS3‐activated genes were also activated by FLS2, while nearly 80% of FLS3‐repressed genes were also repressed by FLS2. Of the top 20 Biological Process categories (False Discovery Rate < 0.05) for each treatment, 13 categories were shared between flg22^Pst^ and flgII‐28^Pst^ in the ED Zone, including “Response to Biotic Stimulus”, “Response to Other Organism”, and “Biological Processes Involved in the Interspecies Interaction Between Organisms,” (Table S2). Consistent with PTI response in other species, transcripts associated with cell wall and cytoskeleton organisation were downregulated in response to both MAMPs in the ED Zone (“Cell Wall Organisation or Biosynthesis”) (Wang et al. 2022). Genes uniquely upregulated in response to flgII‐28^Pst^ were primarily attributed to changes in metabolism (“Cellular Amino Acid Metabolic Proc,” “Alpha‐Amino Acid Metabolic Proc”, “Sulphur Compound Metabolic Proc”, etc.) (Table S2). Of the 335 FLS3‐specific upregulated genes, flgII‐28^Pto^ perception initiated the exclusive transcription of two Ethylene‐Responsive Transcription Factors (ERFs) *Solyc05g051200* and *Solyc09g066350* as compared to two distinctly upregulated ERFs (*Solyc04g012050* and *Solyc06g068830*) and a number of ethylene receptors upon flg22^Pst^ perception. Thus, although SlFLS2 and FLS3 had different coreceptor and MAPK requirements the overall downstream transcriptional responses were similar.

**Figure 5:**
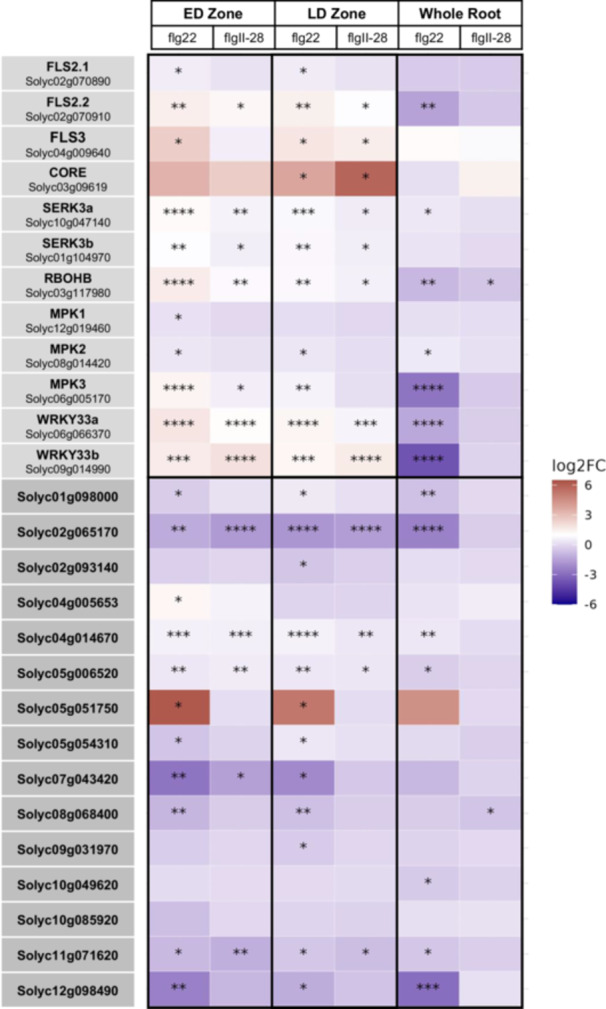
Expression of H7996 genes from the RNAseq data set that encode for proteins directly associated with the PTI signalling pathway as well as PTI‐marker gene candidates from Yu et al. (2021). The colours of the graph represent the Log2FC, while significance is shown through the *p*‐adj values: < 0.05*, 0.0001**, 0.0000001***, 1 × 10^−^^10^ ****. [Color figure can be viewed at wileyonlinelibrary.com]

To better understand immune gene expression, we focused on 12 genes commonly associated with PTI (Gómez‐Gómez and Boller 2000; Hind et al. 2016; Peng and Kaloshian et al. 2014; Wang et al. 2016; Willmann et al. 2014; Zhou et al. 2020; Zipfel et al. 2004) (Figure 5). After flg22^Pst^ treatment, 11 of the 12 were differentially expressed in the LD and ED zone samples, but only seven in the whole root. Similarly, after flgII‐28^Pst^ treatment, a higher number of PTI‐associated genes were differentially expressed in the LD and ED zones but only one in the whole root (Figure 5). Occasionally, a gene showed differential expression in the LD or ED zone compared to the whole root. For example, five genes (*FLS2.2*, *MPK3*, and *RbohB, and WRKY33A/B*) were significantly downregulated in whole root samples while they were significantly upregulated in ED samples in response to flg22^Pst^ and flgII‐28^Pst^ (Figure 5). *CORE* was significantly upregulated in the LD zone, but not in the ED zones of flg22 and flgII‐28‐treated H7996 roots. This was at first surprising given the ED‐specific csp22 ROS burst in H7996 (Figure 1C,D). However, *CORE* is also upregulated in whole roots of H7996 after treatment with *Ralstonia solanacearum* (Meline et al. 2023), suggesting that MAMPs induce expression of this PRR. We hypothesise that the basal levels of *CORE* gene expression are higher in the ED zone and lead to the csp22‐induced ROS burst in that zone, and that CORE is upregulated throughout the LD and whole root after MAMP treatment.

We then evaluated fifteen potential PTI marker genes identified in a previous proteomic analysis (Yu et al. 2021). Upon comparison of the gene expression levels within our whole‐root data, a single gene (*Solyc08g068400*) was significantly repressed in the whole root samples treated with flgII‐28^Pst^ and seven were significant for whole root samples treated with flg22^Pst^ (Figure 5). In contrast, ED zones treated with flgII‐28^Pst^ showed significant differential expression for five of the 15 candidate PTI marker genes and treatment with flg22^Pst^ resulted in significant differential expression for 11 of the 15 candidates in the ED zone. All 5 DEGs from flgII‐28^Pst^ treatment were found within the flg22^Pst^ DEGs. The LD and ED zone shared differential expressions of 10 of the candidate marker genes. Two additional candidates (*Solyc02g065170* and *Solyc09g031970*) were identified in flg22^Pst^ treated LD samples, and only one gene (*Solyc04g005653*) was found in the ED zone but not the LD zone. Together, these results support our understanding of increased immune responses upon MAMP recognition in the root and identify five candidate PTI marker genes (*Solyc02g065170, Solyc04g014670, Solyc05g006520, Solyc07g043420, Solyc11g071620*) for both proteomic and transcriptomic studies.

### 3.5 Early Root Growth Inhibition Is MAMP Dependent but Not RBOHD Dependent

Approximately 20% of the FLS3‐regulated genes were specific to FLS3 (Figure 4A). To test whether the variation in gene expression resulted in different phenotypic outcomes, we tested the impact of each peptide on root growth. Prolonged flg22 exposure leads to early root growth inhibition (Gómez‐Gómez and Boller 2000), and our whole root transcriptomic data revealed that a large number of DEGs related to growth pathways were downregulated for flg22^Pst^‐treated roots but not flgII‐28^Pst^ (Figure S8C). For example, the GO categories “cell cycle” and “cell wall organisation or biogenesis” were enriched among downregulated genes only in flg22^Pst^‐treated roots (Figure S8). Thus, we hypothesised that the robust transcriptional response in the tomato root may have an observable phenotypic outcome after transient exposure to flg22^Pst^.

**Figure 6:**
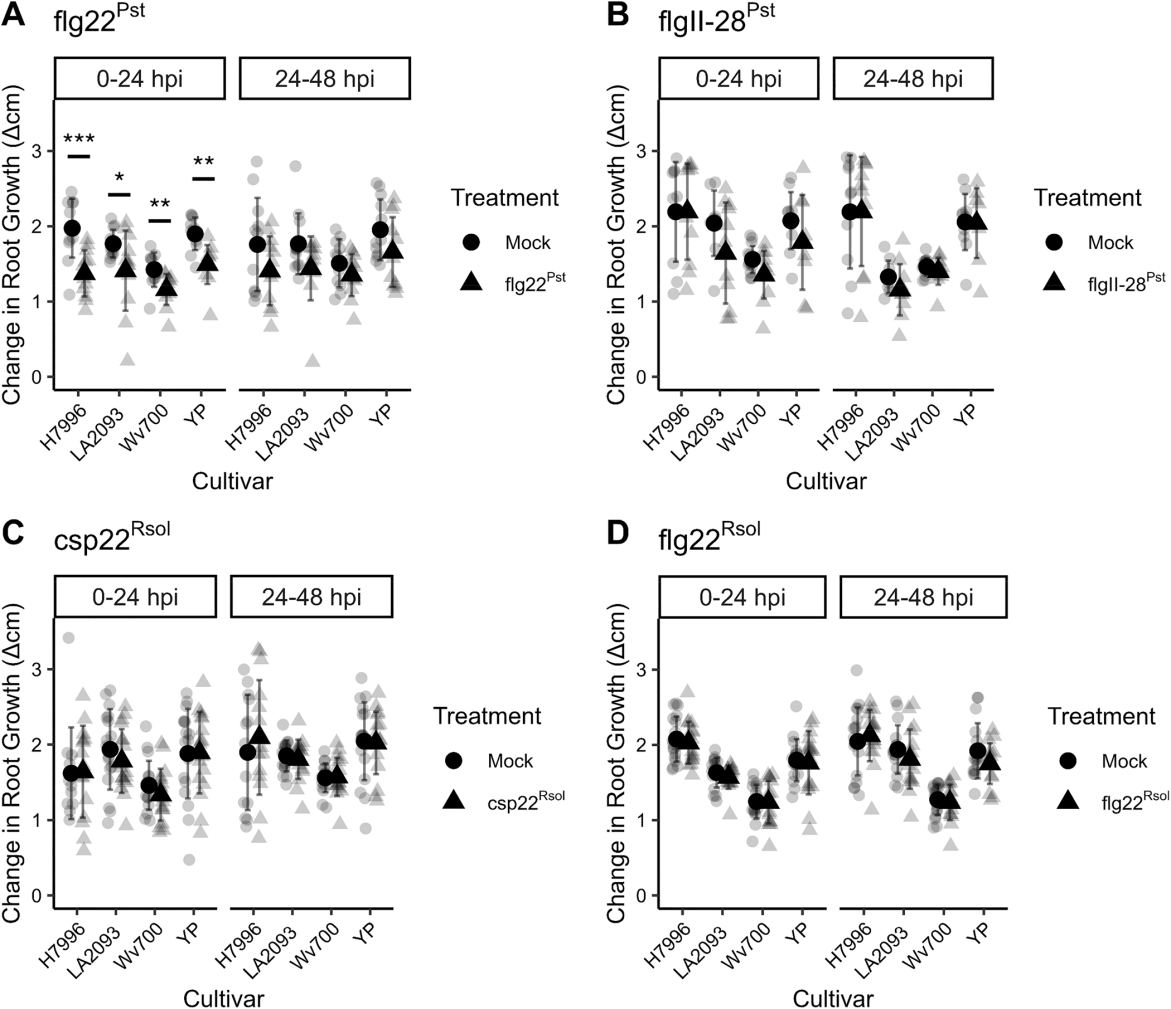
Temporary root growth inhibition is observed for flg22^Pst^ treatment, but not flgII‐28^Pst^, csp22^Rsol^ or flg22^Rsol^. Change in root growth (cm/24 h) for tomato roots of accessions H7996, LA2093, Wv700, and Yellow Pear from 0 to 24 h and 24–48 h. Tomato seedlings treated with (A) 1 µM flg22^Pst^ or mock (water), (B) 100 nM flgII‐28^Pst^ or mock (water), (C) 1 µM csp22^Rsol^ or mock (water), and (D) 1 µM flg22^Rsol^ or mock (water). Values represent the mean ± SD from at least 12 roots per treatment (Wilcoxon, *p < 0.05, **p < 0.01, ***p < 0.001, ****p < 0.0001).

Upon a single treatment with flg22^Pst^ root growth in each of the four accessions tested was temporarily inhibited for the first 24 h postinoculation (hpi) but recovered to that of mock by 48 hpi (Figure 6A). In contrast, treatments of both flgII‐28^Pst^ and csp22^Rsol^ on all four accessions failed to elicit temporary growth inhibition at both 24 and 48 hpi (Figure 6B,C). As a MAMP control, roots were treated with flg22^Rsol^ (Figure 6D); LA0716 was used as a genetic control for csp22^Rsol^ (Figure S3B). The absence of temporary root growth inhibition for flgII‐28^Pst^ and csp22^Rsol^ strengthens our hypothesis that downstream elements of PTI for FLS2, FLS3 and CORE are independent yet overlapping.

To test whether this same temporary growth inhibition and recovery occurred in other FLS2‐mediated PTI events, we performed a root growth assessment on Arabidopsis (Col‐0) seedlings with the same single MAMP flood treatment. Notably, a temporary FLS2‐mediated root growth inhibition for flooded Arabidopsis seedlings did not occur until 48 h posttreatment (Figure S9). Similar to tomato, the Arabidopsis seedlings resumed normal growth rates just 24 h later. Overall, our experiments indicate that root growth inhibition to a single MAMP treatment varies among elicitors, and tomato root response and recovery to a single flg22^Pst^ elicitation occurs more rapidly than that of Arabidopsis.

**Figure 7:**
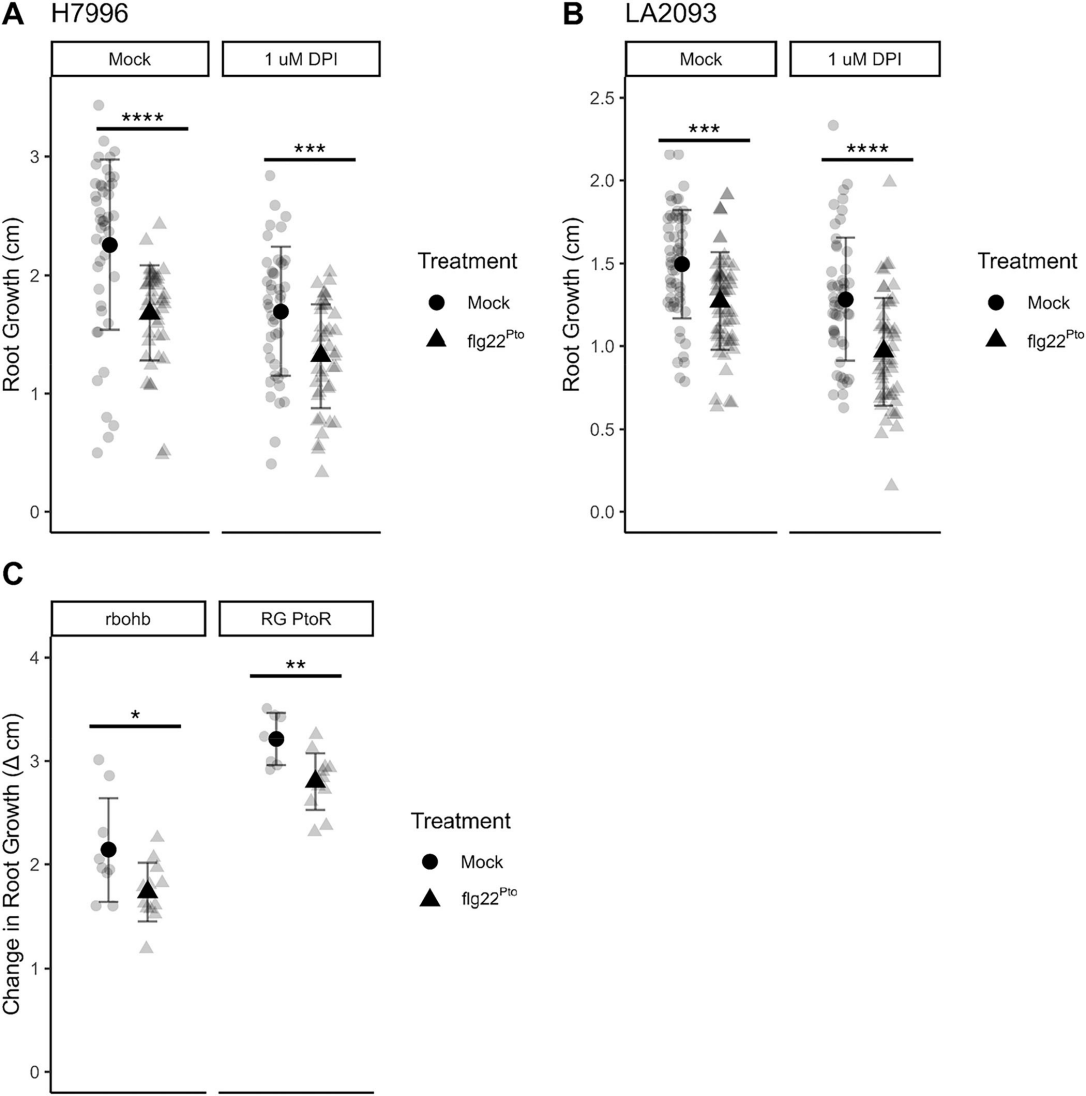
Temporary root growth inhibition is independent of ROS burst in tomato root PTI response. Change in root growth (cm/24 h) for tomato from 0 to 24 h and 24‐48 h postinoculation (hpi). Five‐day‐old tomato seedlings of (A) H7996 and (B) LA2093 were treated with 1 µM DPI or mock (water) 4 h before 1 µM flg22^Pst^ or mock (water) treatment. Values represent the mean ± SD from at least 36 replicates per treatment. (Wilcoxon, *p < 0.05, **p < 0.01, ***p < 0.001, ****p < 0.0001). Five‐day‐old tomato seedlings of (C) rbohb and background Rio Grande PtoR were treated with 1 µM flg22^Pst^ or mock (water) treatment. Values represent the mean ± SD from at least 36 replicates per treatment. (Student's t‐test, *p < 0.05, **p < 0.01, ***p < 0.001, ****p < 0.0001).

MAMP‐induced prolonged RGI is independent of the NADPH oxidase *At*RBOHD (Lu et al. 2009; Shinya et al. 2014; Tran et al. 2020). To examine whether ROS production and temporary RGI are independent, we performed RGI assays using the NADPH oxidase inhibitor diphenyleneiodonium chloride (DPI) alongside flg22^Pst^ treatment for tomato accessions H7996 and LA2093. We first identified the minimum concentration of DPI needed to fully inhibit the ROS burst response (Figure S10). Using this concentration (1 µM), we pretreated tomato seedlings with either DPI or a mock solution before applying flg22^Pst^. Despite the DPI treatment, temporary root growth inhibition was still observed in both H7996 and LA2093 (Figure 7A,B), suggesting that temporary RGI was not dependent on NADPH‐produced ROS.

We repeated the initial single‐treatment growth inhibition experiment on Rio Grande seedlings with a point mutation in *SlRbohB*, leading to a frameshift in exon 1 (Figure S11A). The *rbohb* line displayed an abolishment in ROS response upon treatment with 100 nM flg22 (Figure S11B). Upon treatment with 1 μM flg22^Pst^, the *rbohb* lines and their Rio Grande background parental line exhibited temporary RGI at 24 h compared to mock treatment similar to that of our DPI‐treated roots (Figure 7C). Together, these results suggest that temporary RGI and ROS burst function independently in FLS2‐mediated PTI.

## 4 Conclusions

Pattern‐triggered immunity plays a crucial role in the innate immune response of plants where it is activated by the recognition of conserved microbial patterns through PRRs. These receptors have been successfully transferred within and among species, showing promise in broad‐spectrum resistance strategies for crop protection. However, to effectively engineer crops for broad‐spectrum resistance, we must first understand how each PRR functions in its plant of origin.

We hypothesised that molecular signalling components downstream of Solanum‐specific PRRs *FLS3* and *CORE* in roots would diverge from those of the broadly conserved *FLS2* receptor. The results supported our hypothesis and showed that the signalling pathways in tomato roots downstream of FLS2, FLS3 and CORE diverge in unexpected ways, such as (1) the lack of MPK3 phosphorylation after FLS3 activation compared to FLS2 activation, (2) lack of any MPK phosphorylation with CORE activation and (3) SERK3a and SERK3b co‐receptors differentially control ROS burst upon FLS2 and FLS3 activation. Further, although 80% of the transcriptional responses in the ED zone were similar between FLS2 and FLS3, temporary root growth inhibition was observed after flg22 treatment but not flgII‐28 or csp22. These results support the idea that taxon‐specific PRRs may show more divergence in their signalling pathways compared to well‐conserved PRRs and may be good candidates to enhance resistance within a genus (Snoeck et al. 2024). Further work aimed at understanding the specifics of these pathways will be particularly important for engineering resistance in crops for pathogens that are not natural pathogens of Arabidopsis, such as *Ralstonia solanacearum*.

Additionally, our results show that individual LRR‐RLKs initiated distinct but overlapping PTI responses in tomato roots, with the strongest activity in the early differentiation zone. This was true from the earliest events of perception, including the involvement of co‐receptors, initiation of ROS burst, phosphorylation of MPKs, and transcriptional reprogramming. This is consistent with previous findings that root immune responses are compartmentalised and influenced by developmental stage (Tsai et al. 2023; Chuberre et al. 2018, Üstüner et al. 2022). One challenge in engineering crops with additional resistance traits is that such plants often have growth defects (Ning et al. 2017; Gao et al. 2024; Üstüner et al. 2022). Future work will test the hypothesis that targeted expression of PRRs in the root ED zone can overcome growth‐defence trade‐offs and generate enhanced resistance to soilborne pathogens.

## Conflicts of Interest

The authors declare no conflicts of interest.

## Supporting information


**Fig. S1:** Reactive Oxygen Species (ROS) burst dynamics vary by MAMP type for tomato whole roots. **Fig. S2:** ED‐specific Reactive Oxygen Species Burst is found in additional accessions of tomato for csp22^Rsol^. **Fig. S3:** LA0176 does not respond to csp22^Rsol^. **Fig. S4:** Treatment with different concentrations of peptides still results in ED‐specific ROS in tomato roots. **Fig. S5:** Rio Grande responds to flg22, flgII‐28, and csp22. **Fig. S6:** MAPK phosphorylation at additional timepoints. **Fig. S7:** Top 20 GO Biological Processes categories represented by genes upregulated in response to MAMP treatments in tomato late and early differentiation zones. **Fig. S8:** Top 20 GO Biological Processes categories represented by genes upregulated in response to MAMP treatments in tomato whole roots. **Fig. S9:** Temporary root growth inhibition is observed in *Arabidopsis* seedlings for flg22^Pto^ treatment at 24 hpi, but not earlier. **Fig. S10:** Determination of DPI concentration sufficient to fully inhibit H7996 ROS burst in response to flg22^Pto^. **Fig. S11:** Tomato *rbohb* lines do not respond to flg22.

**Table S1:** Primers and Constructs used in this study.

**Table S2:** GO Biological Function categories represented by genes upregulated in response to MAMP treatments in tomato roots.

## Data Availability

The data that support the findings of this study are available from the corresponding author upon reasonable request.
