# Supplementary material for: Tomato Roots Exhibit Development‐Specific Responses to Bacterial‐Derived Peptides
Source: Plant Cell Environ. 2025 Sep 5;48(12):8771–87. doi: 10.1111/pce.70164 (PMC12586909; doi:10.1111/pce.70164)

Figure S1. Reactive Oxygen Species (ROS) burst dynamics vary by MAMP type for tomato whole roots. Whole root samples of 5-day-old tomato seedlings of H7996, LA2093, Wv700, and Yellow Pear were treated with (**A**) 1 μM flg22^{Pst} or mock (water), (**B**) 1 μM flg22^{Rsol} or mock (water), (**C**)1 μM csp22^{Rsol} or mock (water), and (**D**)100 nM flgII-28^{Pst} or mock (water). Values in Figure 1C, 1E, and 1G represent the mean +SD from at least 18 replicates per treatment (Student's t-test, *p<0.05, **p<0.01, ***p<0.001, ****p<0.0001). Whole root samples of H7996 were treated with (**E**) 1 μM flg22^{Pst} , 100nM flgII-28^{Pst} or mock (water), or (**F**) 1 μM csp22^{Rsol} or mock (water). Values represent the mean ± SD from at least 6 replicates per treatment. The assay was repeated three times with similar results.

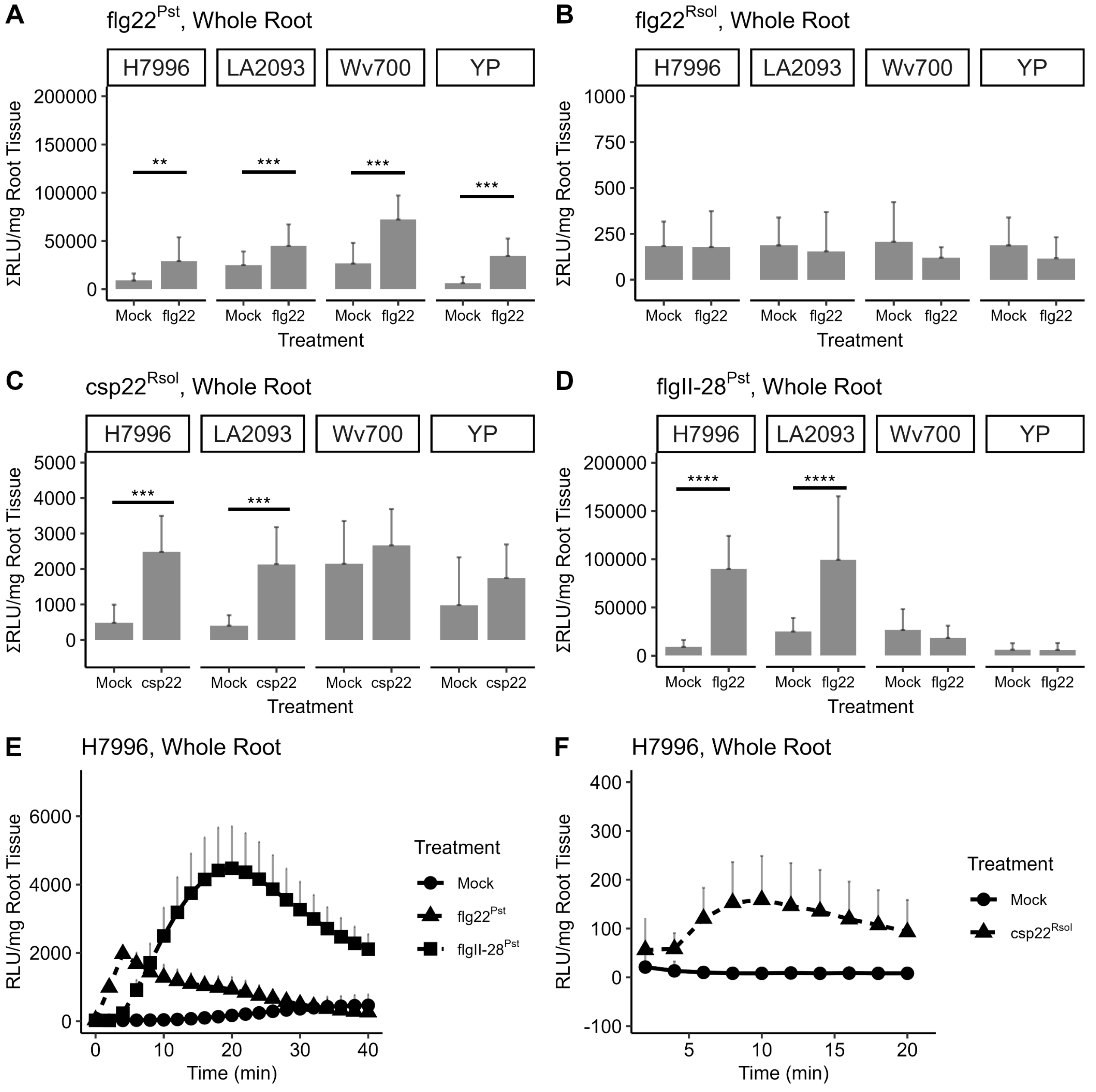

Figure S2. Reactive Oxygen Species Burst is primarily found in the Early Differentiation Zone for additional cultivars of tomato. (**A**) Schematic representation of the root zones, including the Late Differentiation Zone, Early Differentiation Zone, and Meristematic/Transition Zone. 5-day-old tomato seedlings of (**B**) Ailsa Craig, (**C**) Rutgers, (**D**) Black from Tula, or (**E**) Brandywine accessions were divided into the root Late Differentiation and Early Differentiation Zone; LD or ED samples were treated with1 μM csp22^{Rsol} or mock (water). Values represent the mean ± SD from at least 6 replicates per treatment.

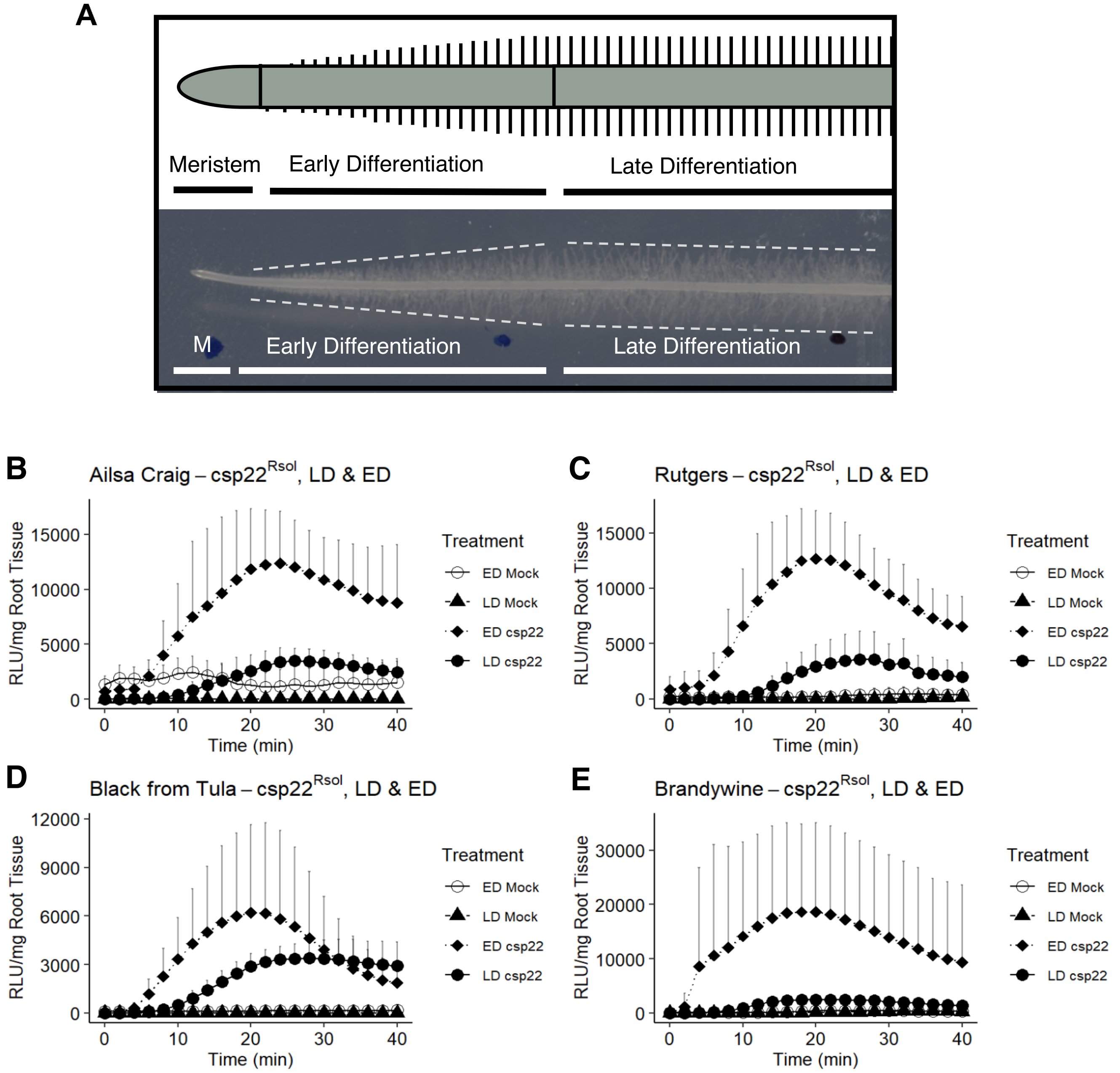

Figure S3. LA0176 does not respond to csp22^{Rsol} . (A) Change in root growth (cm/24 hour) for tomato roots of LA0176 from 0-24 hours and 24-48 hours. Tomato seedlings treated with 1 µM csp22^{Rsol} or mock (water). Values represent the mean ±SD from at least 18 replicates per treatment. (Student's t-test, *p<0.05, **p<0.01, ***p<0.001, ****p<0.0001) (B) Root samples from 5-day-old tomato seedlings of LA0716 were treated with 1 µM csp22^{Rsol} or mock (water). Values represent the mean ± SD from at least 6 replicates per treatment. The experiment was repeated three times with similar results.

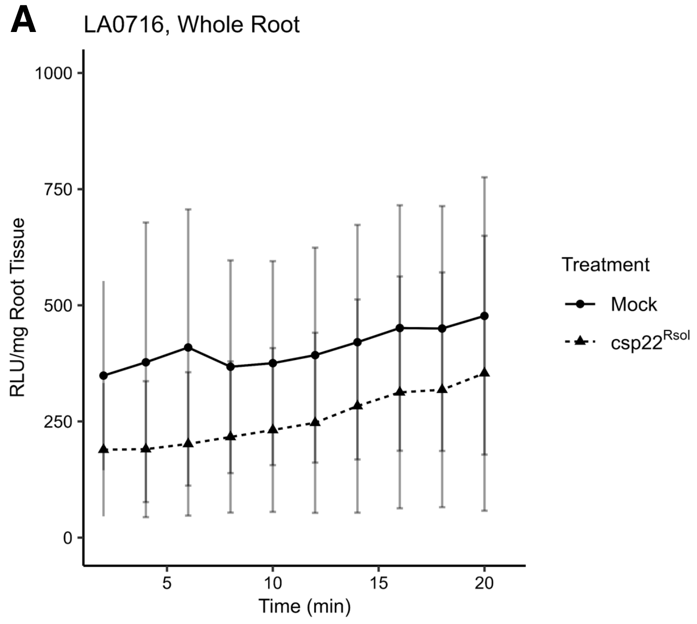

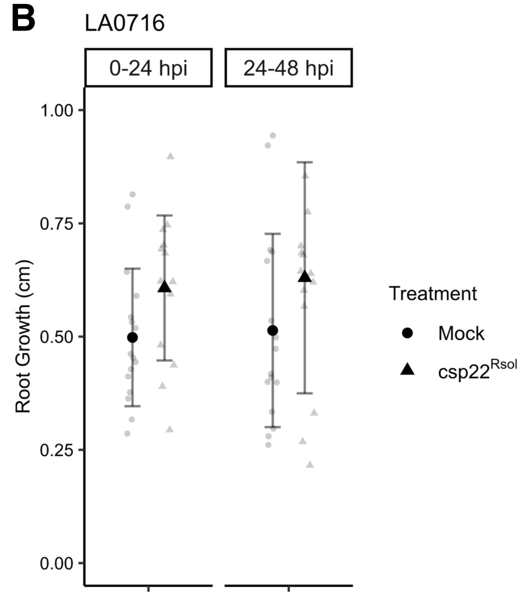

Figure S4. Treatment with different concentrations of peptides still results in ED-specific ROS in tomato roots. (A) H7996 and LA2093 treated with 100 nM flgII-28^{Pst}, (B) H7996 and LA2093 treated with 1 uM flgII-28^{Pst}. Values represent the mean ± SD from at least 6 replicates per treatment. The assay was repeated three times with similar results.

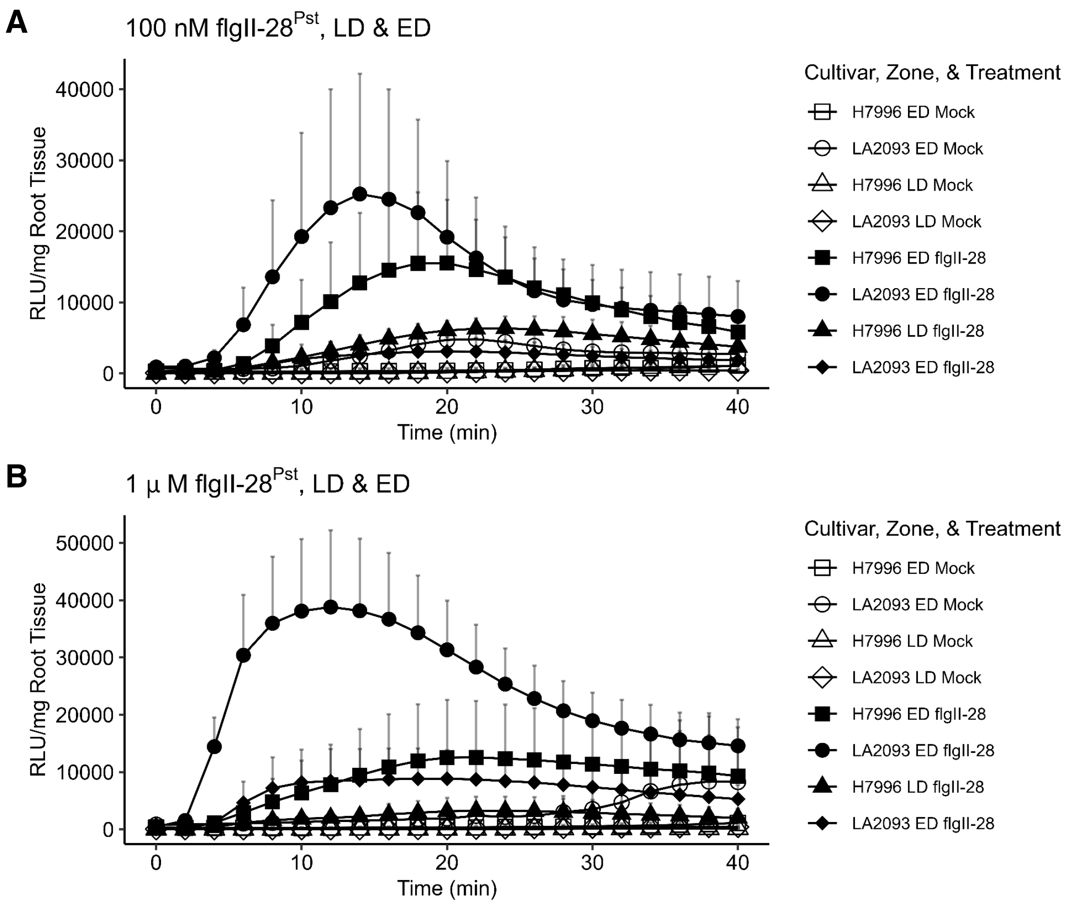

Figure S5. Rio Grande responds to flg22, flgII-28, and csp22. Root samples from 5-day-old tomato seedlings of Rio Grande were treated with 1 µM flg22^{Pst}, 100 nM flgII-28^{Pst}, 1 µM flgII-28^{Pst} , or mock treatment. Values represent the mean ± SD from at least 6 replicates per treatment. The experiment was repeated two times with similar results.

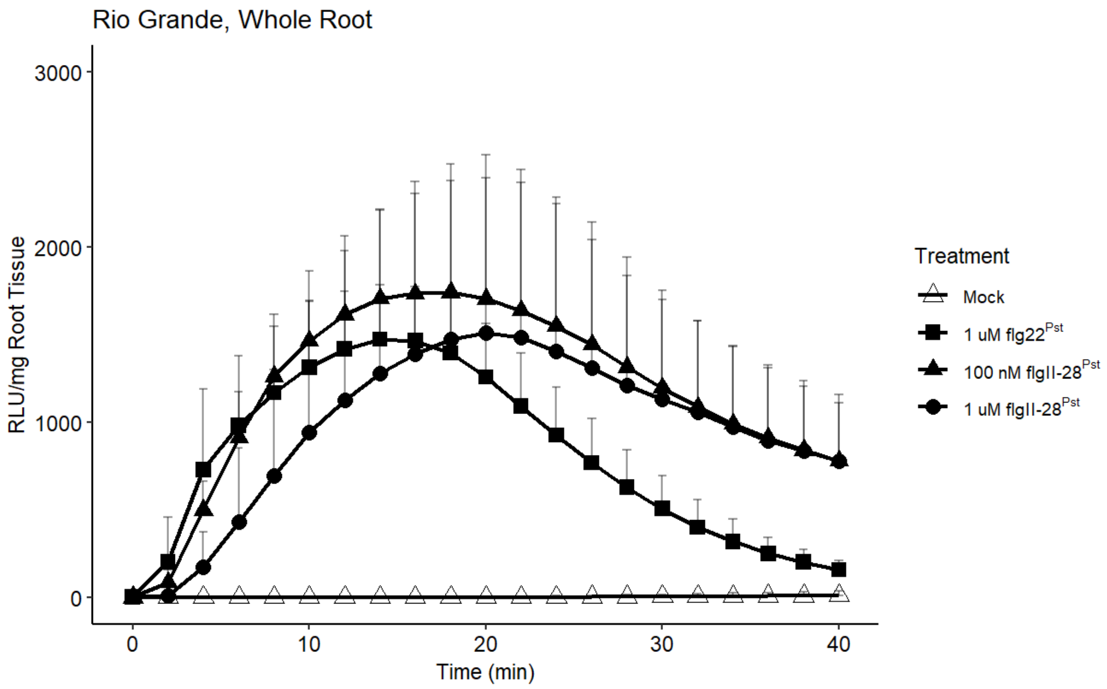

Figure S6. Temporal Dynamics of MAPK phosphorylation in tomato leaf and root tissues of H7796 upon treatment with PAMPs. (A) Leaf samples treated with mock (water), 1 μM flg22, or 1μM csp22 at 10-, 20-, or 30-minutes post PAMP treatment. (B) Root sections treated with mock (water), 1μM flg22, or 1μM csp22 at 10- or 20-minutes post PAMP treatment. LD = Late Differentiation Zone; ED = Early Differentiation Zone. Phosphorylation was assessed by western blot using Phospho-ERK1/2 HRP-linked antibody (CellSignaling, #8544). Total proteins were detected by Anti-Actin HRP-linked Antibody (Abbkine). A Bradford assay was also used for equal protein loading. The assay was repeated three times with similar results.

A

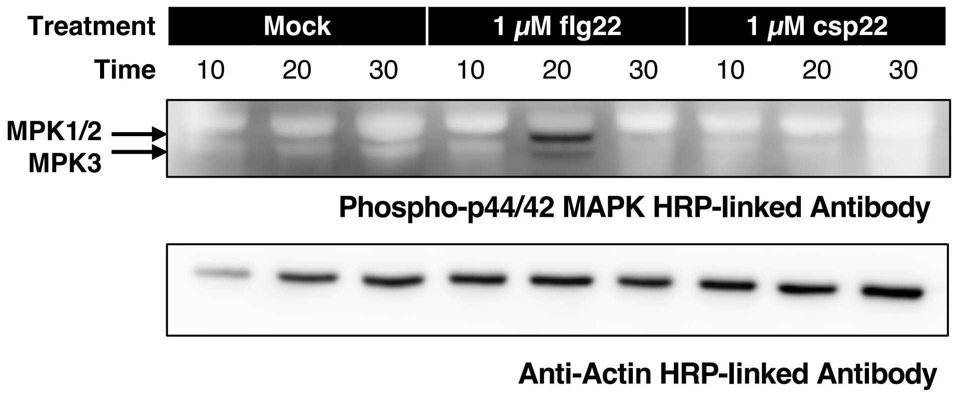

B

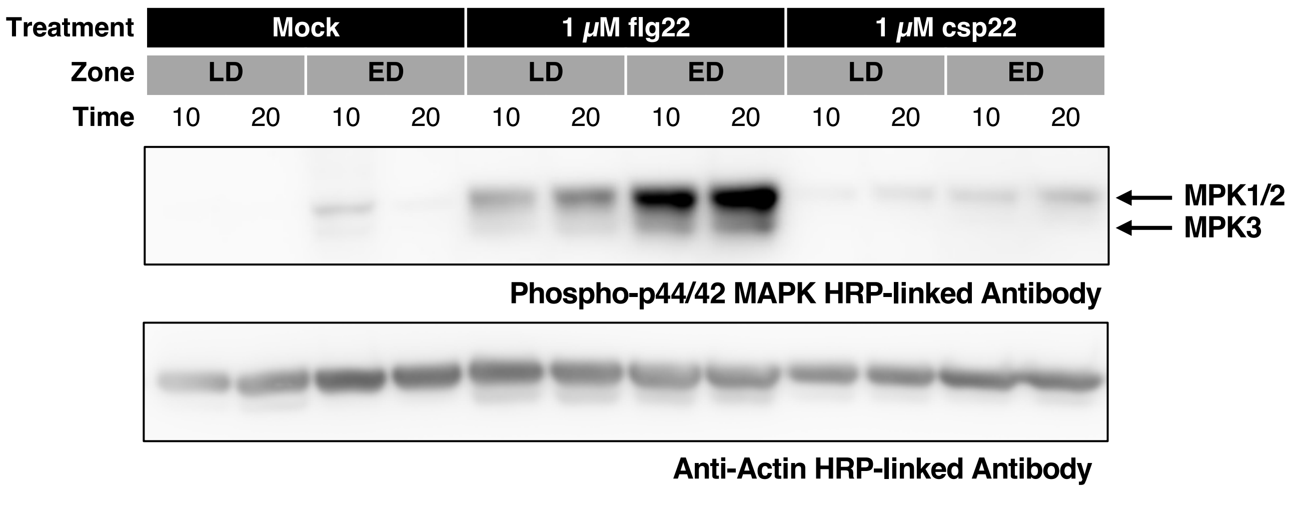

Figure S7. Top 20 GO Biological Processes categories represented by genes upregulated in H7996 response to MAMP treatments in tomato late and early differentiation zones. Blue triangles represent the –log10(FDR). Grey bars represent the number of DEGs in the GO Category.

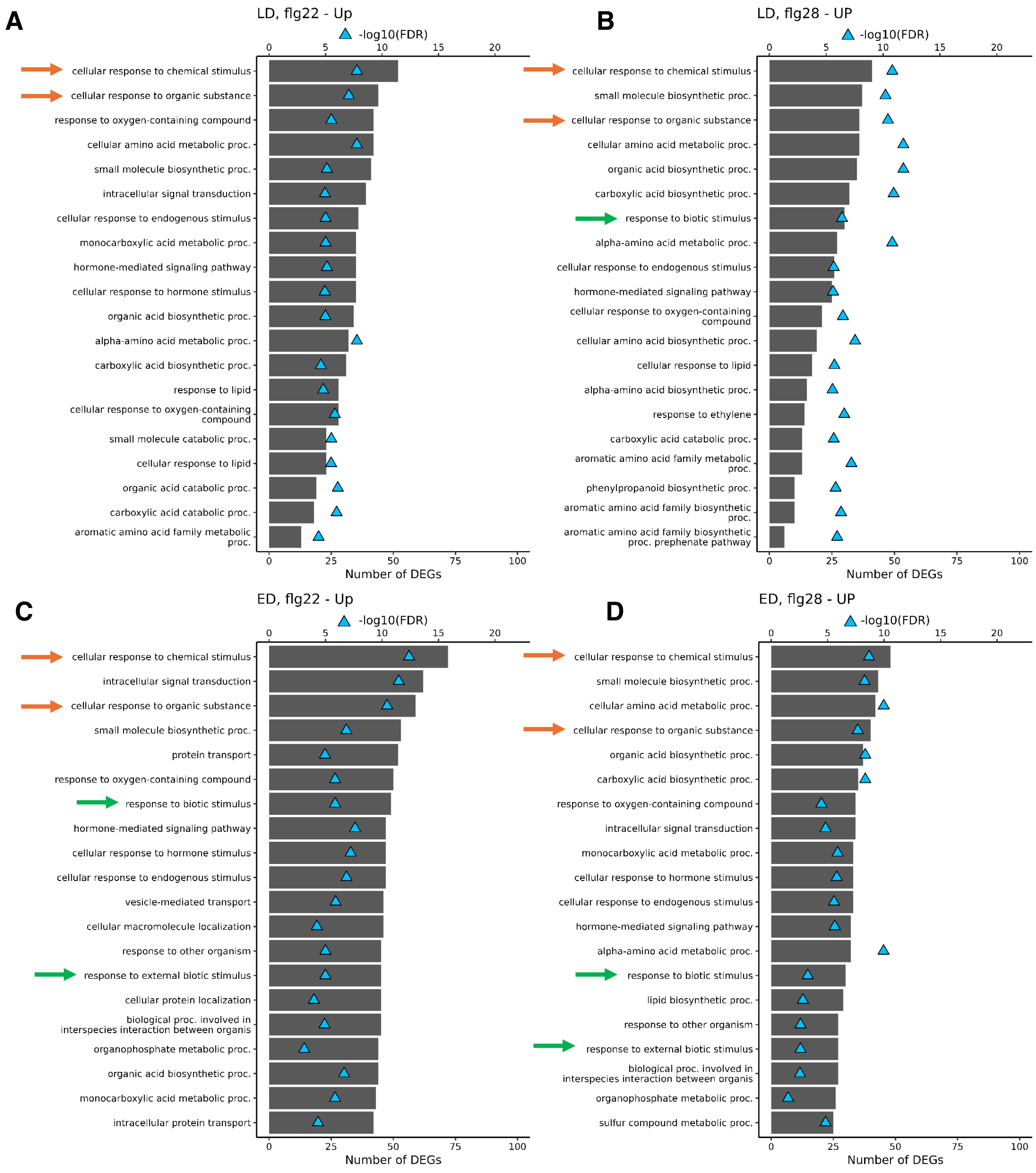

Figure S8. Top 20 GO Biological Processes categories represented by genes upregulated in H7996 response to MAMP treatments in tomato whole roots. Blue triangles represent the –log10(FDR). Grey bars represent the number of DEGs in the GO Category.

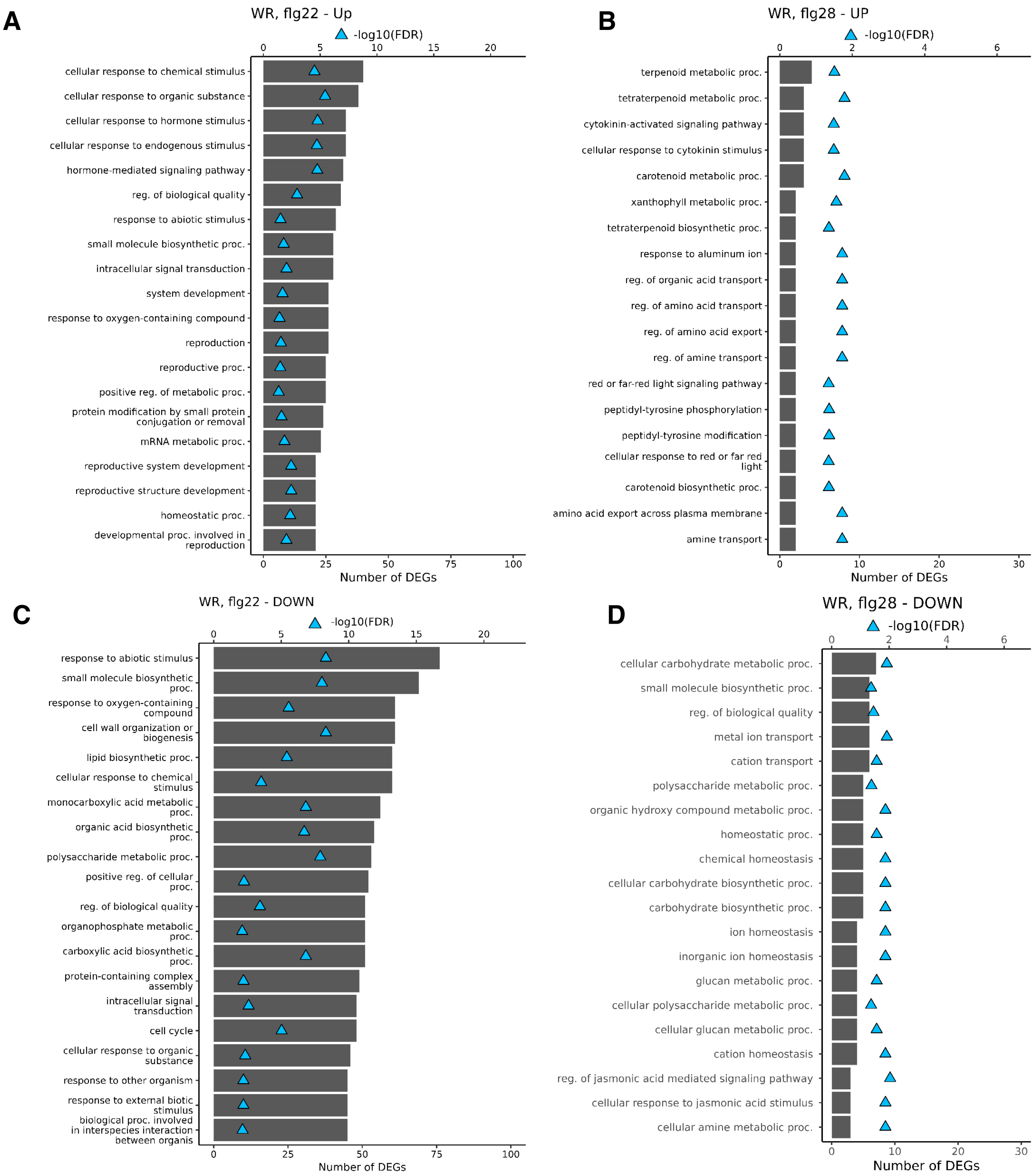

Figure S9. Temporary root growth inhibition is observed in Arabidopsis seedlings for flg22Pto treatment at 24 hpi, but not earlier. Change in root growth (cm/24 hour) for Arabidopsis roots of from 0-24 hours, 24-48 hours, and 48-72 hours post inoculation. Arabidopsis (A) Col-0 seedlings treated with 1 μM flg22^{Pst} or mock (water). Values represent the mean ±SD from at least 18 roots per treatment (Wilcoxon, *p<0.05, **p<0.01, ***p<0.001, ****p<0.0001).

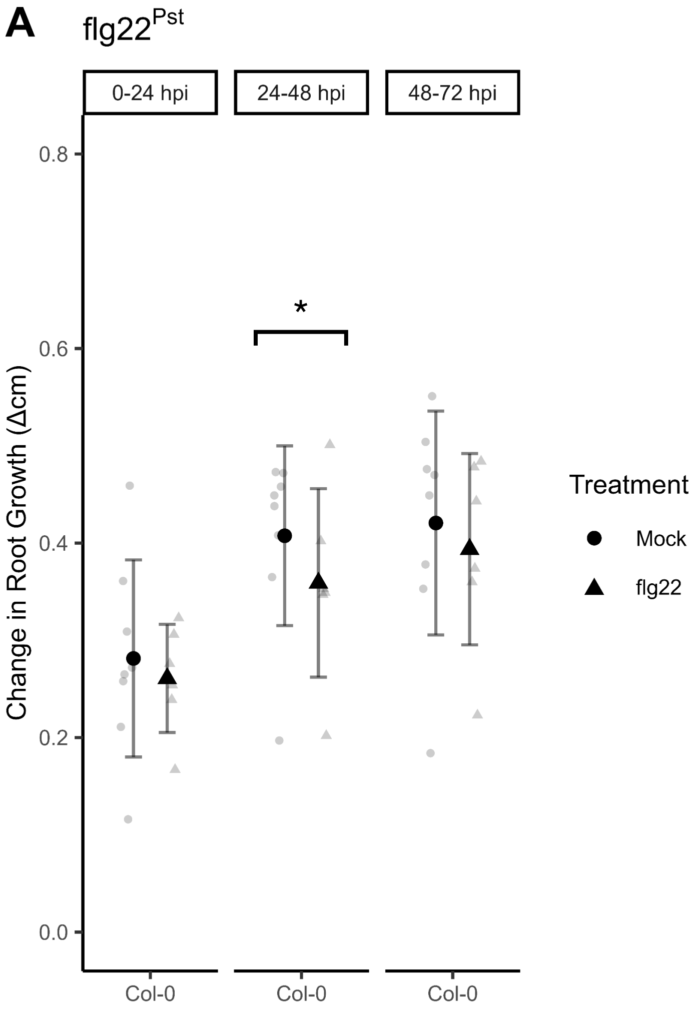

Figure S10. Determination of DPI concentration sufficient to fully inhibit H7996 ROS burst in response to flg22^{Pst}. Root samples from 5-day-old tomato seedlings of H7996 were treated with 0.2 μM DPI, 0.5 μM DPI, 1 μM DPI, or mock (water) for four hours prior to 1 μM flg22^{Pst} treatment. Values represent the mean ± SD from at least 6 replicates per treatment. The experiment was repeated two times with similar results.

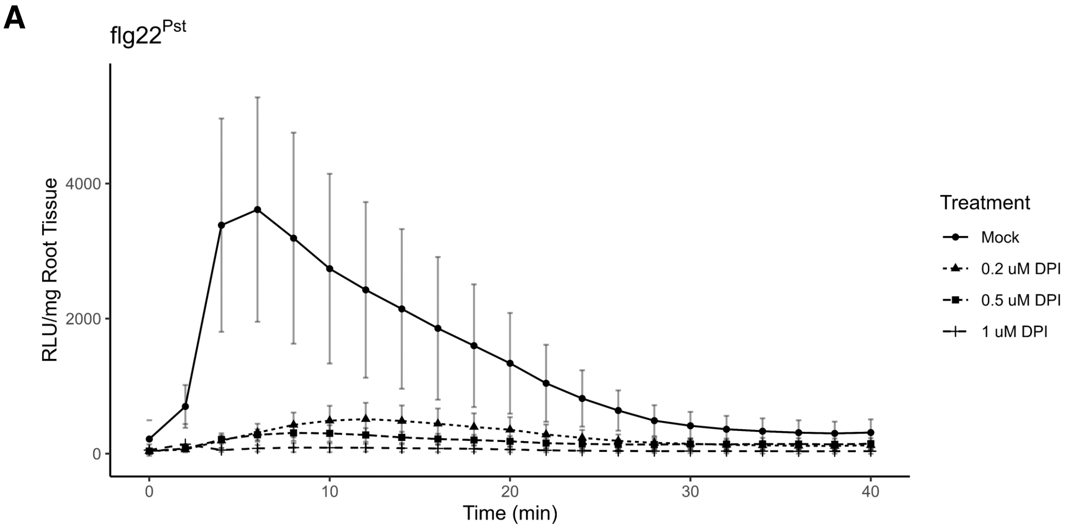

Figure S11. Development of *rbohb* mutant line using CRISPR-cas9 displayed abolished ROS production. (**A**) Gene structure of RBOHB (Solyc03g117980) with the region targeted for editing denoted in red. Below: Sanger sequence of mutant line compared to wild-type Rio Grande PtoR tomato. (**B**) ROS production in response to 100 nM flg22. Each experiment included two plants with four leaf disks per plant. The assay was independently repeated two times with similar results. Error bars = SEM.

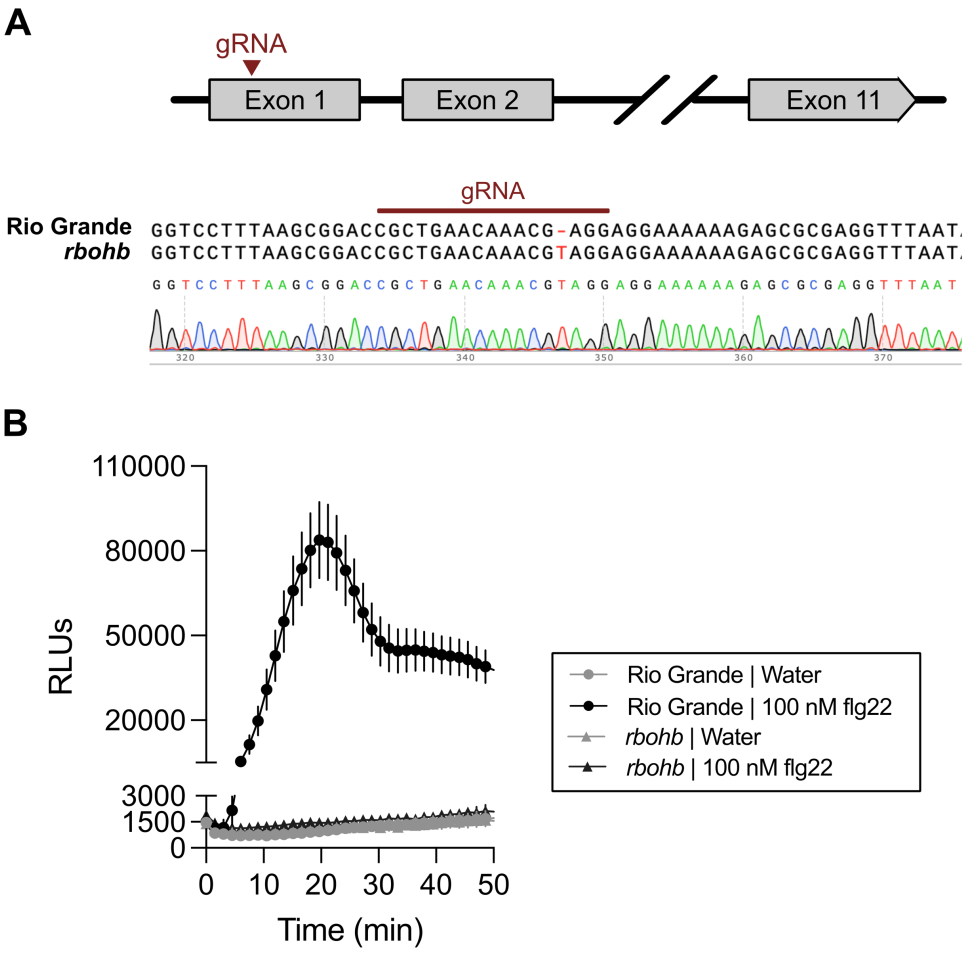

Supplement: Supplementary file 1 — Fig. S1: Reactive Oxygen Species (ROS) burst dynamics vary by MAMP type for tomato whole roots. Fig. S2: ED‐specific Reactive Oxygen Species Burst is found in additional accessions of tomato for csp22Rsol. Fig. S3: LA0176 does not respond to csp22Rsol. Fig. S4: Treatment with different concentrations of peptides still results in ED‐specific ROS in tomato roots. Fig. S5: Rio Grande responds to flg22, flgII‐28, and csp22. Fig. S6: MAPK phosphorylation at additional timepoints. Fig. S7: Top 20 GO Biological Processes categories represented by genes upregulated in response to MAMP treatments in tomato late and early differentiation zones. Fig. S8: Top 20 GO Biological Processes categories represented by genes upregulated in response to MAMP treatments in tomato whole roots. Fig. S9: Temporary root growth inhibition is observed in Arabidopsis seedlings for flg22Pto treatment at 24 hpi, but not earlier. Fig. S10: Determination of DPI concentration sufficient to fully inhibit H7996 ROS burst in response to flg22Pto. Fig. S11: Tomato rbohb lines do not respond to flg22. [file PCE-48-8771-s001.pdf]
